# Prognostic impact of HER2-low expression in triple-negative breast cancer of high-grade special histological type and no special type

**DOI:** 10.1371/journal.pone.0325715

**Published:** 2025-06-13

**Authors:** Claudia Grosse, Petar Noack, Alexandra Grosse, Heike Kathleen Schwarz, Peter Schrenk, Heike Frauchiger-Heuer, Rupert Langer, Zsuzsanna Varga

**Affiliations:** 1 Department of Pathology and Molecular Pathology, Johannes Kepler University Linz, Kepler University Hospital GmbH, Linz, Austria; 2 Pathology Institute Enge, Zurich, Switzerland; 3 Department of Hematology and Oncology, Johannes Kepler University Linz, Kepler University Hospital GmbH, Linz, Austria; 4 Department of General and Visceral Surgery, Johannes Kepler University Linz, Kepler University Hospital GmbH, Linz, Austria; 5 Department of Gynecology, University Hospital Zurich, Zurich, Switzerland; 6 Department of Pathology and Molecular Pathology, University Hospital Zurich, Zurich, Switzerland; Gangnam Severance Hospital, Yonsei University College of Medicine, KOREA, REPUBLIC OF

## Abstract

Interest in HER2-low breast cancer has grown in recent years due to advancements in novel anti-HER2 antibody-drug conjugates. This study examined the impact of HER2-low expression on survival outcomes in triple-negative breast cancer (TNBC) of high-grade special histological type (ST) and no special type (NST) and investigated the prognostic significance of TNBC subtype (high-grade ST vs. NST) within HER2 0 and HER2-low expression subgroups. Clinicopathological and survival data of 504 patients with stage I-III TNBC, with or without neoadjuvant chemotherapy (NAC), were analyzed, including 400 patients with TNBC NST and 104 patients with high-grade TNBC ST. HER2-low status was not identified as an independent prognostic factor for survival in the overall cohort, nor within the high-grade TNBC ST and TNBC NST subgroups. Among patients who did not receive NAC, TNBC subtype (high-grade ST vs. NST) was independently associated with DDFS and DFS in the HER2 0 subgroup, but not in the HER2-low subgroup. Patients with HER2 0 high-grade TNBC ST exhibited significantly worse OS (*p* = 0.008), DDFS (*p* < 0.001), and DFS (*p* < 0.001) compared to those with HER2 0 TNBC NST. Among patients with either HER2 0 or HER2-low tumors treated with NAC, no significant survival difference was observed between high-grade TNBC ST and TNBC NST. These findings suggest that the prognostic impact of TNBC subtype (high-grade ST vs. NST) on survival outcomes may be modulated by HER2 status in a subset of TNBC patients.

## Introduction

With the advent of new anti-HER2 antibody-drug conjugates (ADCs) and other targeted agents, along with promising results from clinical trials testing novel agents in the HER2 setting, recognizing HER2-low expression in breast cancer (BC) (defined as HER2 immunohistochemistry [IHC] score of 1+ or 2+ without *HER2* gene amplification) is becoming increasingly significant [1–6]. HER2-low status is more common in hormone receptor (HR)-positive BC [7–11], but it is also observed in triple-negative BC (TNBC), with reported prevalence rates ranging from 16.2% to 39.3% [8–10,12–14]. To date, the biology of TNBC with HER2-low expression remains poorly understood [13], and current literature shows conflicting results regarding the prognostic implications of HER2-low status in HR-negative BC. While some studies indicate poorer survival outcomes in patients with HER2 0 TNBC compared to those with HER2-low TNBC, others have found no significant survival differences between HER2 0 and HER2-low HR-negative BC [7–22].

Previous studies on the prognostic impact of HER2-low expression in TNBC have primarily focused on comparing survival outcomes between HER2-low and HER2 0 tumors, without stratifying TNBC into prognostically relevant subgroups. Triple-negative BC is a heterogeneous group of malignancies that includes both TNBC of no special type (NST) and a variety of special histological types (ST). Based on their biological behavior and clinical course, TNBC ST can be classified into low-grade and high-grade lesions and neoplasms of uncertain biological potential [23–29]. Recent research has shown that high-grade TNBC ST is associated with significantly worse survival outcomes compared to TNBC NST among patients with unilateral, non-metastatic BC treated with primary surgery [30]. However, the prognostic implications of HER2-low expression in high-grade TNBC ST and TNBC NST subgroups, as well as the impact of HER2-low status on the prognostic significance of TNBC subtype (ST high-grade vs. NST) remain unclear.

The primary objective of this study was to evaluate the impact of HER2-low expression and its association with clinicopathological characteristics and survival outcomes in a cohort of patients with non-metastatic high-grade TNBC ST and TNBC NST. The secondary aim was to analyze the prognostic significance of TNBC subtype (high-grade ST vs. NST) in HER2-low and HER2 0 expression subgroups.

## Materials and methods

### Patients and data evaluation

All consecutive patients with histologically confirmed unifocal, unilateral, stage I-III high-grade TNBC ST or TNBC NST who underwent primary surgery or neoadjuvant chemotherapy (NAC) followed by surgery at the University Hospital Zurich (UHZ), Switzerland, or Kepler University Hospital (KUH), Austria, between 2010 and 2023 were included in this dual-center study. Patients with metastatic disease at diagnosis, synchronous or metachronous HR-positive BC or multiple primary TNBCs were excluded. The clinicopathological characteristics extracted from pathology reports and medical files included patient age at diagnosis, prognostic TNBC subtype (high-grade ST vs. NST), clinical (cT) and pathological (pT) tumor stage, clinical (cN) and pathological (pN) nodal stage, histological tumor grade, HR/HER2 status, Ki-67 proliferation index (determined by visual estimation of the percentage of Ki-67-positive tumor cells), surgery type (breast-conserving therapy or mastectomy), treatment type (neoadjuvant, adjuvant), and treatment modality (chemotherapy, immunotherapy/targeted therapy, radiotherapy). TNBC samples with < 10% of estrogen receptor (ER) or progesterone receptor (PR)-positive tumor cells were classified as ER- and PR-negative. In TNBC cases treated with NAC, HR/HER2 status, Ki-67 proliferation index, and histological tumor grade were assessed using pre-treatment core needle biopsy reports, and the Residual Cancer Burden (RCB) score and pathological complete response (pCR) were also recorded. Pathological complete response was defined as the absence of residual invasive cancer in the breast and regional lymph nodes, with or without residual carcinoma in situ (ypT0/ypTis ypN0). RCB was determined based on the primary tumor bed size, the overall tumor cellularity, the number of lymph nodes and the size of largest lymph node metastasis, using the MD Anderson RCB calculator [31]. HER2 status was assessed by HER2 IHC and fluorescence in situ hybridization (FISH) analysis. HER2 IHC was performed using the 4B5 rabbit monoclonal antibody on a Ventana Benchmark Ultra autostainer (Hoffmann-La Roche Ltd, Basel, Switzerland) in the KUH cohort as well as the anti-HER2 clone CB11 (Hoffmann-La Roche Ltd, Basel, Switzerland) on a Ventana Benchmark semiautomated staining system with Ventana reagents (2010−2011) and the anti-HER2 clone 4B5 (Hoffmann-La Roche Ltd, Basel, Switzerland) on a fully automated Leica Bond autostainer (Leica Biosystems, Nunningen, Switzerland) (2011−2023) in the UHZ cohort. HER2 IHC was evaluated using the time-current American Society of Clinical Oncology/College of American Pathologists (ASCO/CAP) guidelines for HER2 scoring [32,33]. For FISH analysis, a dual fluorescence kit (PathoVysion, Vysis, Abbott Ltd, Diagnostic Division Baar, Switzerland) was used at the UHZ to analyze the *HER2* gene on 2-µm thick paraffin sections. At the KUH, all specimens were analyzed using the Ventana HER2 Dual ISH Assay (Hoffmann-La Roche Ltd, Basel, Switzerland) on 4-µm thick paraffin sections. Tumors with HER2 IHC scores of 0 were classified as HER2-negative, while those with HER2 IHC scores of 1+ or 2+ and a negative *HER2* gene amplification result via ISH techniques were classified as HER2-low. High-grade special histological types of TNBC included high-grade metaplastic carcinomas (spindle cell carcinoma, squamous cell carcinoma, matrix-producing metaplastic carcinoma, and metaplastic carcinoma with mixed metaplastic elements), high-grade salivary gland-like tumors (such as solid-basaloid adenoid-cystic carcinoma), invasive lobular carcinoma (ILC, triple-negative subtype), mixed-type NST-ILC (triple-negative subtype), polymorphous carcinoma, micropapillary carcinoma, and high-grade encapsulated papillary carcinoma [23–29]. The adapted survival indicators were overall survival (OS), distant disease-free survival (DDFS), and disease-free survival (DFS). OS was defined as the time from diagnosis to death from any cause or last follow-up. DDFS was defined as the time from diagnosis to distant tumor recurrence or last follow-up; and DFS was the time from diagnosis to any tumor recurrence (distant or locoregional), last follow-up, or death from any cause. The data were extracted from a previously published patient database [30] and accessed between February 1, 2023, and June 12, 2023 (KUH), and between June 12, 2023, and June 30, 2023 (UHZ). The study was approved by the Ethics Committee of the Johannes Kepler University Linz, Austria, (IRB number: 1040/2024), and the Ethics Committee of the Canton of Zurich, Switzerland, (KEK-2012-553/PB2019/001111).

### Statistical analysis

Percentages were used to describe categorical variables, and means ± standard deviations, medians, and ranges were used to describe continuous variables. Categorical variables were compared using the chi-square test or Fisher’s exact test, continuous variables were analyzed using the Student’s t-test. The Kaplan-Meier method was used to estimate survival probabilities, and survival distributions between groups were compared using the log-rank test. Case matching was performed to eliminate the effects of differences in pT category on survival outcomes in the non-NAC HER2 0 high-grade TNBC ST and TNBC NST subgroups. The Cox proportional hazards model was used for multivariate analysis of all variables with a *p*-value < 0.20 in univariate analysis, and adjusted hazard ratios (HRs) with 95% confidence intervals (95% CIs) were calculated. Statistical analyses were performed using SPSS Statistics software (version 28.0, SPSS, Chicago, IL, USA), and the threshold for statistical significance was set at *p*-value < 0.05.

## Results

### Clinicopathological characteristics

#### Patient and tumor characteristics.

Of the 504 patients included in the study (UHZ: n = 209; KUH: n = 295), 104 (20.6%) had high-grade TNBC ST, and 400 (79.4%) had TNBC NST. The majority of high-grade TNBC ST were high-grade metaplastic carcinomas (n = 67, 64.4%), followed by high-grade salivary gland-like tumors (n = 12, 11.5%), ILC (n = 10, 9.6%), high-grade encapsulated papillary carcinomas (n = 10, 9.6%), and other histological subtypes (n = 5, 4.8%) (Table 1). Patient and tumor characteristics are summarized in Table 2. The median age at diagnosis was 54.0 years (mean: 54.8 ± 15.6 years, 95% CI: 53.4–56.1; range: 16–93 years). 310 (61.5%) patients underwent primary surgery (NST: n = 230, 45.6%; high-grade ST: n = 80, 15.9%), and 194 (38.5%) patients (NST: n* *= 170, 33.7%; high-grade ST: n = 24, 4.8%) received NAC, including 22 high-grade TNBC ST patients and 164 TNBC NST patients for whom follow-up information were available. NAC regimens for the 22 high-grade TNBC ST patients with follow-up data included anthracycline/cyclophosphamide/taxane (n = 14) with additional platinum/pembrolizumab in 3 cases, additional platinum in 3 cases and additional capecitabine/platinum in 1 case; anthracycline/taxane (n = 4) with additional platinum in 2 cases; taxane (n = 2) with a PARP-inhibitor in 1 case; platinum/etoposide/ifosfamide (n = 1), and anthracycline/cyclophosphamide (n = 1) (S1 Table). 15 (68.2%) of the 22 patients with high-grade TNBC ST who received NAC underwent adjuvant chemotherapy, most commonly with capecitabine (n = 11), in combination with pembrolizumab in 2 cases and with platinum/taxane in 1 case (S1 Table). Among the 164 NAC-treated TNBC NST patients with available follow-up data, the majority received neoadjuvant anthracycline/cyclophosphamide/taxane-based chemotherapy regimens (n = 128), with additional platinum/pembrolizumab in 23 cases, additional platinum in 19 cases and additional 5-fluorouracil in 2 cases (S2 Table). 44 (26.8%) of the 164 NAC-treated TNBC NST patients received adjuvant chemotherapy (capecitabine: n = 34; other: n = 10), including 4 patients who underwent adjuvant (chemo-)immunotherapy (capecitabine/pembrolizumab: n = 4). Overall, 23 (14.0%) of the 164 TNBC NST patients received neoadjuvant (chemo-)immunotherapy with pembrolizumab, and 26 (15.9%) received adjuvant immunotherapy/targeted therapy (pembrolizumab: n = 22; PARP inhibitor: n = 3; sacituzumab-govitecan: n = 1) (S2 Table). Among the 22 NAC-treated high-grade TNBC ST patients with available follow-up data, 4 (18.2%) received neoadjuvant (chemo-)immunotherapy/targeted therapy (pembrolizumab: n = 3; PARP-inhibitor: n = 1), and 3 (13.6%) received adjuvant immunotherapy/targeted therapy (pembrolizumab: n = 2; PARP-inhibitor: n = 1) (S1 Table).

**Table 1:** Histological subtypes of high-grade TNBC ST (n = 104).

Histological subtypes	N (%)
Metaplastic carcinoma high-grade	67 (64.4)
Matrix-producing	30 (28.8)
Squamous cell carcinoma	15 (14.4)
Mixed metaplastic elements	14 (13.5)
Spindle cell carcinoma	8 (7.7)
Salivary gland-like tumor high-grade	12 (11.5)
Adenoid-cystic carcinoma, solid-basaloid	12 (11.5)
Encapsulated papillary carcinoma high-grade	10 (9.6)
With invasion	8 (7.7)
With coexistent NST-ILC	2 (1.9)
ILC (TN subtype)	10 (9.6)
Micropapillary carcinoma	2 (1.9)
Mixed type NST-ILC	2 (1.9)
Polymorphous carcinoma	1 (1.0)

TNBC, triple-negative breast cancer; ST, special type; NST, no special type; ILC, invasive lobular carcinoma.

**Table 2:** Correlations between clinicopathological features and TNBC subtype in HER2 0 and HER2 1 + /2 + subgroups (n = 504).

	HER2 0 TNBC				HER2 1 + /2 + TNBC			
Variable	Overall (n = 333)	NST (n = 260)	ST high-grade (n = 73)		Overall (n = 171)	NST (n = 140)	ST high-grade (n = 31)	
	N (%)	N (%)	N (%)	*p*-Value	N (%)	N (%)	N (%)	*p*-Value
**Age group** (years)								
< 50	149 (44.7)	126 (48.5)	23 (31.5)	**0.010**	65 (38.0)	56 (40.0)	9 (29.0)	0.255
≥ 50	184 (55.3)	134 (51.5)	50 (68.5)		106 (62.0)	84 (60.0)	22 (71.0)	
**Mean age** (years)	53.8 ± 15.8	52.7 ± 15.2	57.6 ± 17.3	**0.019**	56.7 ± 15.1	56.0 ± 15.6	59.8 ± 12.1	0.200
**Year of diagnosis**								
2010-2017	201 (60.4)	156 (60.0)	45 (61.6)	0.800	90 (52.6)	73 (52.1)	17 (54.8)	0.786
2018-2023	132 (39.6)	104 (40.0)	28 (38.4)		81 (47.4)	67 (47.9)	14 (45.2)	
**c/pT category**								
T1	137 (41.1)	113 (43.5)	24 (32.9)	**0.019**	76 (44.4)	65 (46.4)	11 (35.5)	0.535
T2	147 (44.1)	116 (44.6)	31 (42.5)		77 (45.0)	61 (43.6)	16 (51.6)	
T3/T4	49 (14.7)	31 (11.9)	18 (24.7)		18 (10.5)	14 (10.0)	4 (12.9)	
**c/pN category**								
N0	205 (61.6)	158 (60.8)	47 (64.4)	0.605	119 (69.6)	93 (66.4)	26 (83.9)	0.146
N1/N1mi	95 (28.5)	74 (28.5)	21 (28.8)		37 (21.6)	34 (24.3)	3 (9.7)	
N2/N3	33 (9.9)	28 (10.8)	5 (6.8)		15 (8.8)	13 (9.3)	2 (6.5)	
**Nodal status** (post-NAC)								
N-	246 (73.9)	195 (75.0)	51 (69.9)	0.377	132 (77.2)	106 (75.7)	26 (83.9)	0.327
N+	87 (26.1)	65 (25.0)	22 (30.1)		39 (22.8)	34 (24.3)	5 (16.1)	
**Ki-67 index (%)**								
≤ 20	14 (4.2)	5 (1.9)	9 (12.3)	**<0.001**	10 (5.8)	4 (2.9)	6 (19.4)	**<0.001**
> 20	319 (95.8)	255 (98.1)	64 (87.7)		161 (94.2)	136 (97.1)	25 (80.6)	
**Mean Ki-67 index** (%)	59.9 ± 21.8	61.8 ± 20.8	53.0 ± 24.0	**0.002**	61.3 ± 21.8	64.0 ± 20.4	49.1 ± 24.1	**<0.001**
Grade*								
G2	25 (7.7)	19 (7.3)	6 (9.2)	0.603	20 (12.0)	13 (9.3)	7 (25.9)	**0.015**
G3	300 (92.3)	241 (92.7)	59 (90.8)		147 (88.0)	127 (90.7)	20 (74.1)	
**NAC**								
Yes	129 (38.7)	113 (43.5)	16 (21.9)	**<0.001**	65 (38.0)	57 (40.7)	8 (25.8)	0.122
No	204 (61.3)	147 (56.5)	57 (78.1)		106 (62.0)	83 (59.3)	23 (74.2)	
**NAI/NATT** (missing: 7)								
Yes	15 (4.5)	15 (5.8)	0 (0.0)	**0.036**	12 (7.2)	8 (5.9)	4 (12.9)	0.172
No	315 (95.5)	243 (94.2)	72 (100.0)		155 (92.8)	128 (94.1)	27 (87.1)	
**Surgery type**								
BCT	208 (62.5)	172 (66.2)	36 (49.3)	**0.009**	106 (62.0)	86 (61.4)	20 (64.5)	0.749
Mastectomy	125 (37.5)	88 (33.8)	37 (50.7)		65 (38.0)	54 (38.6)	11 (35.5)	
**Adjuvant CT** (missing: 22)								
Yes	195 (61.3)	146 (57.9)	49 (74.2)	**0.015**	101 (61.6)	78 (58.6)	23 (74.2)	0.109
No	123 (38.7)	106 (42.1)	17 (25.8)		63 (38.4)	55 (41.4)	8 (25.8)	
**Adjuvant RT** (missing: 22)								
Yes	257 (80.8)	203 (80.6)	54 (81.8)	0.817	116 (70.7)	96 (72.2)	20 (64.5)	0.398
No	61 (19.2)	49 (19.4)	12 (18.2)		48 (29.3)	37 (27.8)	11 (35.5)	
**Adjuvant IT/TT** (missing: 22)								
Yes	23 (7.2)	22 (8.7)	1 (1.5)	**0.044**	13 (7.9)	10 (7.5)	3 (9.7)	0.689
No	295 (92.8)	230 (91.3)	65 (98.5)		151 (92.1)	123 (92.5)	28 (90.3)	

TNBC, triple-negative breast cancer; ST, special type; NST, no special type; NAC, neoadjuvant chemotherapy; NAI, neoadjuvant immunotherapy; NATT, neoadjuvant targeted therapy; BCT, breast conserving therapy; CT, chemotherapy; RT, radiotherapy; IT, immunotherapy; TT, targeted therapy. *No grading according to WHO 2019 in adenoid-cystic carcinoma.

#### HER2 expression and clinicopathological associations.

**Fig 1:**
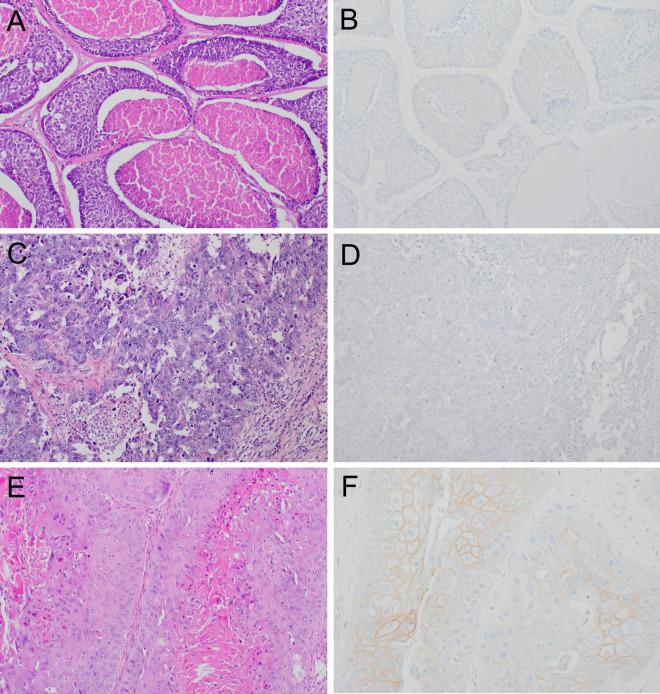
Histological subtypes and HER2 IHC scores of high-grade TNBC ST. **(A, B)** Adenoid cystic carcinoma, solid-basaloid variant, with HER2 IHC score 0. **(C, D)** High-grade encapsulated papillary carcinoma with HER2 IHC score 0. **(E, F)** High-grade metaplastic squamous cell carcinoma with HER2 IHC score 2 + /ISH non-amplified.

**Fig 2:**
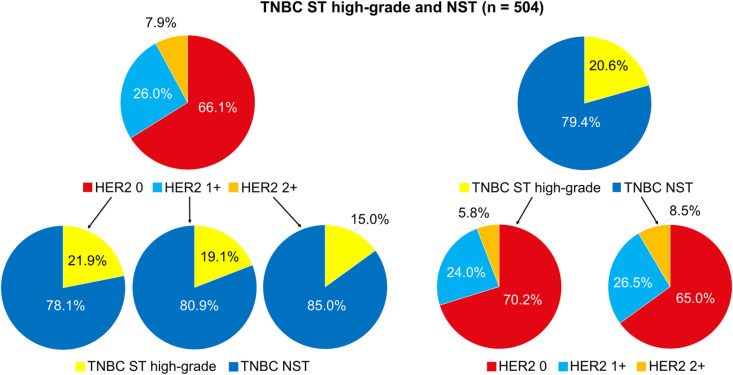
Distribution of HER2 IHC scores across TNBC subgroups. TNBC triple-negative breast cancer, ST special type, NST no special type.

Among the 504 TNBC samples, 333 (66.1%) were classified as HER2 IHC score 0, 131 (26.0%) as HER2 IHC score 1 + , and 40 (7.9%) as HER2 IHC score 2+ (Figs 1 and 2). The majority of both high-grade TNBC ST (70.2%) and TNBC NST (65.0%) were classified as HER2 IHC score 0, with no significant difference in HER2 expression between the two groups (*p* = 0.521). In the overall study cohort, which included both NAC-treated and non-NAC patients, individuals with HER2 0 high-grade TNBC ST were significantly older (*p* = 0.010) and exhibited a higher c/pT category (*p* = 0.019), a lower Ki-67 index (*p* < 0.001), a lower rate of NAC administration (p < 0.001), as well as higher rates of mastectomy (*p* = 0.009) and adjuvant chemotherapy (*p* = 0.015) compared to patients with HER2 0 TNBC NST (Table 2). Among patients with HER2-low tumors, those with high-grade TNBC ST displayed a lower Ki-67 index (*p* < 0.001) and a significantly lower (pre-treatment) histological tumor grade (*p* = 0.015) compared to their HER2-low TNBC NST counterparts (Table 2). Furthermore, in the overall cohort, HER2-low high-grade TNBC ST exhibited a significantly lower histological tumor grade than HER2 0 high-grade TNBC ST (*p* = 0.036) (S3 Table). This observation remained consistent in non-NAC-treated patients when comparing HER2-low versus HER2 0 TNBC (both high-grade ST and NST combined) (*p* = 0.019), as well as when comparing HER2-low versus HER2 0 TNBC NST alone (*p* = 0.049) (S4 Table). Among tumors treated with primary surgery, HER2 0 high-grade TNBC ST demonstrated a significantly higher pT category (*p* = 0.038), a lower Ki-67 index (*p* < 0.001), and an increased rate of mastectomy (*p* = 0.040) compared to HER2 0 TNBC NST (S5 Table). In contrast, HER2-low high-grade TNBC ST showed a lower Ki-67 index (*p* = 0.010), but no significant difference in mastectomy rate relative to HER2 0 TNBC NST (S5 Table). Among tumors treated with NAC, the pCR rate was significantly lower and the mean RCB score was significantly higher in high-grade TNBC ST compared to TNBC NST. This pattern was observed both in the overall NAC cohort (pCR: 8.3% vs. 50.6%, *p* < 0.001; RCB: 2.259 ± 1.052 vs. 1.143 ± 1.447, *p* < 0.001) and within the HER2 0 (pCR: *p* = 0.002, RCB: *p* < 0.001) and HER2-low (pCR: *p* = 0.017, RCB: *p* = 0.011) subgroups (S6 Table). In contrast, no significant differences in mean RCB score or pCR rate were observed between HER2 0 and HER2-low high-grade TNBC ST, HER2 0 and HER2-low TNBC NST, or HER2 0 and HER2-low TNBC cases overall (including both high-grade ST and NST) (S7 Table).

### Survival analysis

A total of 385 TNBC NST patients (NAC: n = 164; non-NAC: n = 221; HER2 0: n = 252; HER2 1 + /2 + : n = 133) and 95 high-grade TNBC ST patients (NAC: n = 22; non-NAC: n = 73; HER2 0: n = 64; HER2 1 + /2 + : n = 31) with available follow-up data were included in the survival analysis. The median follow-up duration was 63.0 months (mean: 75.1 ± 55.0, 95% CI: 68.8–81.4) for TNBC patients who did not receive NAC and 39.5 months (mean: 57.3 ± 46.8, 95% CI: 50.6–64.1) for those who were treated with NAC.

### Survival outcomes in non-NAC patients

**Fig 3:**
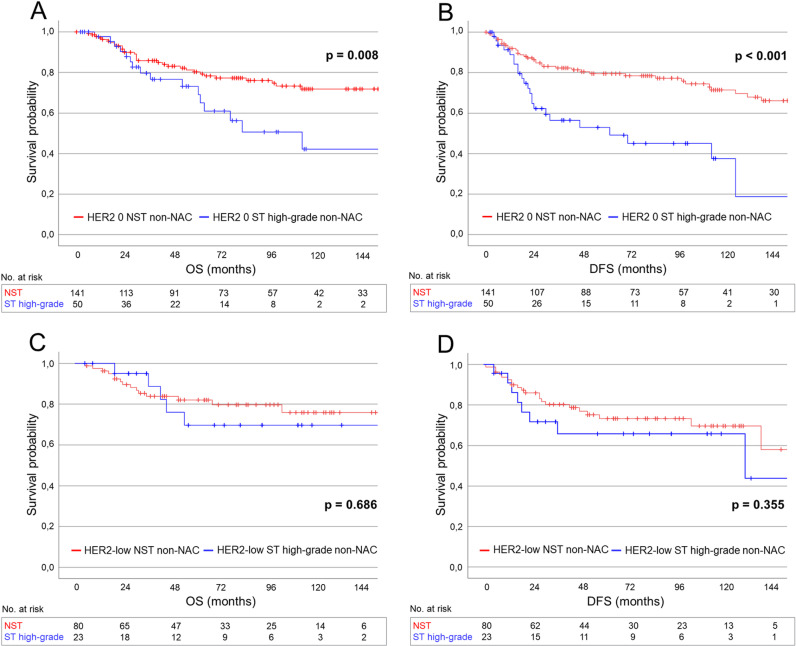
(A, B) Overall survival (A) and disease-free survival (B) in non-NAC patients with HER2 0 high-grade TNBC ST versus those with HER2 0 TNBC NST. (C, D) Overall survival (C) and disease-free survival (D) in non-NAC patients with HER2-low high-grade TNBC ST versus those with HER2-low TNBC NST.

Among patients who underwent primary surgery, the 5-year OS, DDFS, and DFS rates were 82.2% (95% CI: 72.1–85.1), 82.2% (95% CI: 71.8–84.8), and 75.9% (95% CI: 65.7–79.5), respectively, for patients with HER2 0 TNBC, and 82.5% (95% CI: 70.8–88.0), 84.5% (95% CI: 73.5–89.9), and 74.8% (95% CI: 62.4–81.2), respectively, for patients with HER2-low TNBC. No significant differences in survival outcomes were observed between HER2 0 and HER2-low TNBC patients in the overall non-NAC cohort (OS: *p* = 0.398; DDFS: *p* = 0.694; DFS: *p* = 0.754), nor within the TNBC NST (OS: *p* = 0.715; DDFS: *p* = 0.851; DFS: *p* = 0.630) or high-grade TNBC ST subgroups (OS: *p* = 0.149; DDFS: *p* = 0.453; DFS: *p* = 0.169), and HER2 status did not show an independent prognostic association with survival in regression analyses (S8-S10 Tables). Patients with HER2 0 high-grade TNBC ST demonstrated significantly worse OS, DDFS, and DFS compared to those with HER2 0 TNBC NST, both in the overall non-NAC cohort (OS: *p* = 0.008; DDFS: *p* < 0.001; DFS: *p* < 0.001) (Figs 3A and B, S11 Table) and in the pT-matched subgroup of high-grade TNBC ST and TNBC NST patients (OS: *p* = 0.013; DDFS: *p* = 0.006; DFS: *p* < 0.001) (S1 Fig, S12 Table). In contrast, no significant survival differences were observed between HER2-low high-grade TNBC ST and HER2-low TNBC NST patients (OS: *p* = 0.686; DDFS: *p* = 0.071; DFS: *p* = 0.355) (Figs 3C and D). In multivariate analyses, TNBC subtype (high-grade ST vs. NST) was independently associated with DDFS and DFS in the HER2 0 subgroup, but not in the HER2-low subgroup (Tables 3 and 4).

**Table 3:** Univariate and multivariate analyses of clinicopathological variables in non-NAC patients with HER2 0 high-grade TNBC ST and TNBC NST (n = 191).

Univariate	OS			DDFS			DFS		
	HR	95% CI	*p*-Value	HR	95% CI	*p*-Value	HR	95% CI	*p*-Value
**Age** (years)									
< 50	1		**0.045**	1		0.749	1		0.091
≥ 50	1.96	1.02-3.79		1.12	0.56-2.27		1.64	0.92-2.92	
**Year of diagnosis**									
2010-2017	1		0.285	1		0.321	1		0.138
2018-2023	1.48	0.72-3.05		1.45	0.70-3.01		1.58	0.86-2.90	
**TNBC subgroup**									
NST	1		**0.010**	1		**<0.001**	1		**<0.001**
ST high-grade	2.20	1.21-4.00		3.16	1.62-6.19		2.80	1.64-4.78	
**pT category**									
T1	1		**0.003**	1		**0.004**	1		**0.002**
T2	1.59	0.84-3.02		1.62	0.75-3.50		1.70	0.96-3.01	
T3/T4	3.54	1.69-7.39		4.08	1.76-9.48		3.39	1.73-6.66	
**Nodal status**									
N-	1		**<0.001**	1		**<0.001**	1		**<0.001**
N+	2.57	1.47-4.48		3.41	1.76-6.63		2.61	1.58-4.32	
**Ki-67 index** (%)									
≤ 20	1		0.360	1		0.135	1		0.254
> 20	0.58	0.18-1.86		0.41	0.12-1.32		0.55	0.20-1.53	
**Grade**									
G2	1		0.663	1		0.460	1		0.448
G3	0.73	0.18-3.03		0.58	0.14-2.45		0.64	0.20-2.05	
**Adjuvant CT**									
Yes	1		0.400	1		0.286	1		0.104
No	1.33	0.68-2.61		1.51	0.71-3.21		1.62	0.91-2.91	
**Adjuvant RT**									
Yes	1		0.161	1		0.623	1		0.171
No	1.58	0.84-2.98		1.22	0.55-2.69		1.51	0.84-2.71	
**Multivariate**		**OS**			**DDFS**			**DFS**	
	**HR**	**95% CI**	***p*-Value**	**HR**	**95% CI**	***p*-Value**	**HR**	**95% CI**	***p*-Value**
**Age** (years)									
< 50	1		0.191	–	–	–	1		0.564
≥ 50	1.59	0.79-3.18					1.20	0.64-2.25	
**Year of diagnosis**									
2010-2017	–	–	–	–	–	–	1		0.320
2018-2023							1.37	0.74-2.57	
**TNBC subgroup**									
NST	1		0.093	1		**0.007**	1		**0.003**
ST high-grade	1.71	0.91-3.21		2.65	1.31-5.34		2.35	1.34-4.13	
**pT category**									
T1	1		**0.023**	1		0.082	1		**0.043**
T2	1.16	0.60-2.25		1.20	0.54-2.63		1.28	0.71-2.31	
T3/T4	2.85	1.29-6.29		2.69	1.09-6.63		2.51	1.21-5.19	
**Nodal status**									
N-	1		**<0.001**	1		**<0.001**	1		**<0.001**
N+	2.81	1.59-4.98		3.40	1.74-6.66		2.77	1.66-4.63	
**Ki-67 index** (%)									
≤ 20	–	–	–	1		0.738	–	–	–
> 20				0.80	0.22-2.89				
**Adjuvant CT**									
Yes	–	–	–	–	–	–	1		0.192
No							1.52	0.81-2.86	
**Adjuvant RT**									
Yes	1		0.061	–	–	–	1		0.088
No	1.88	0.97-3.62					1.73	0.92-3.26	

TNBC, triple-negative breast cancer; ST, special type; NST, no special type; NAC, neoadjuvant chemotherapy; OS, overall survival; DDFS, distant disease-free survival; DFS, disease-free survival; CT, chemotherapy; RT, radiotherapy.

**Table 4:** Univariate and multivariate analyses of clinicopathological variables in non-NAC patients with HER2-low high-grade TNBC ST and TNBC NST (n = 103).

Univariate		OS			DDFS			DFS	
	HR	95% CI	*p*-Value	HR	95% CI	*p*-Value	HR	95% CI	*p*-Value
**Age** (years)									
< 50	1		0.085	1		0.382	1		0.630
≥ 50	3.62	0.84-15.61		1.75	0.50-6.15		1.23	0.53-2.89	
**Year of diagnosis**									
2010-2017	1		0.555	1		0.882	1		0.624
2018-2023	1.35	0.50-3.65		1.09	0.37-3.17		1.23	0.54-2.78	
**TNBC subgroup**									
NST	1		0.687	1		0.080	1		0.360
ST high-grade	1.23	0.45-3.39		2.42	0.90-6.51		1.47	0.65-3.32	
**pT category**									
T1	1		**0.004**	1		**0.008**	1		**0.004**
T2	20.40	2.70-153.86		7.81	1.74-35.00		3.39	1.42-8.14	
T3/T4	48.16	4.93-470.08		16.41	2.61-103.08		6.69	1.92-23.30	
**Nodal status**									
N-	1		**0.026**	1		**<0.001**	1		**0.006**
N+	2.77	1.13-6.79		5.66	2.10-15.24		2.90	1.36-6.15	
**Ki-67 index** (%)									
≤ 20	1		0.598	1		0.267	1		0.243
> 20	1.72	0.23-12.83		0.48	0.14-1.74		0.53	0.18-1.54	
**Grade**									
G2	1		0.323	1		0.623	1		0.799
G3	2.76	0.37-20.59		0.73	0.21-2.57		0.87	0.30-2.52	
**Adjuvant CT**									
Yes	1		**<0.001**	1		**0.030**	1		**<0.001**
No	5.22	2.17-12.56		3.08	1.12-8.50		3.73	1.78-7.84	
**Adjuvant RT**									
Yes	1		**0.002**	1		0.111	1		**<0.001**
No	4.28	1.70-10.74		2.22	0.83-5.94		3.83	1.75-8.38	
**Multivariate**		**OS**			**DDFS**			**DFS**	
	**HR**	**95% CI**	***p*-Value**	**HR**	**95% CI**	***p*-Value**	**HR**	**95% CI**	***p*-Value**
**Age** (years)									
< 50	1		0.354	–	–	–	–	–	–
≥ 50	2.11	0.44-10.18							
**TNBC subgroup**									
NST	–	–	–	1		0.091	–	–	–
ST high-grade				2.42	0.87-6.74				
**pT category**									
T1	1		**0.002**	1		0.035	1		**0.015**
T2	14.93	1.91-116.54		4.90	1.04-23.0		2.48	1.01-6.08	
T3/T4	67.60	6.45-708.65		12.38	1.82-84.19		6.38	1.76-23.13	
**Nodal status**									
N-	1		0.096	1		**0.002**	1		**0.002**
N+	2.21	0.87-5.63		5.35	1.88-15.20		3.48	1.57-7.75	
**Adjuvant CT**									
Yes	1		0.091	1		0.281	1		0.081
No	2.69	0.85-8.47		2.20	0.53-9.18		2.26	0.91-5.63	
**Adjuvant RT**									
Yes	1		0.061	1		0.486	1		**0.021**
No	3.18	0.95-10.68		1.63	0.41-6.44		3.06	1.19-7.90	

TNBC, triple-negative breast cancer; ST, special type; NST, no special type; NAC, neoadjuvant chemotherapy; OS, overall survival; DDFS, distant disease-free survival; DFS, disease-free survival; CT, chemotherapy; RT, radiotherapy.

#### Survival outcomes in patients receiving NAC.

**Fig 4:**
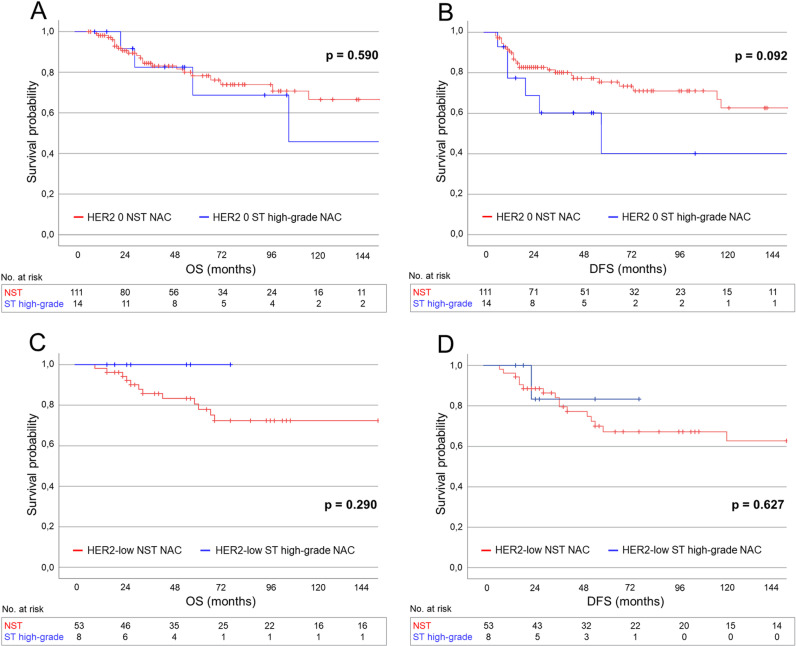
(A, B) Overall survival (A) and disease-free survival (B) in patients with HER2 0 high-grade TNBC ST versus those with HER2 0 TNBC NST receiving neoadjuvant chemotherapy. (C, D) Overall survival (C) and disease-free survival (D) in patients with HER2-low high-grade TNBC ST versus those with HER2-low TNBC NST receiving neoadjuvant chemotherapy.

Among patients treated with NAC, the 5-year OS, DDFS, and DFS rates were 83.2% (95% CI: 68.4–86.0), 80.8% (95% CI: 69.2–85.6), and 76.8% (95% CI: 63.0–81.0), respectively, for patients with HER2 0 TNBC, and 80.3% (95% CI: 61.3–87.1), 78.7% (95% CI: 63.5–87.1), and 73.8% (95% CI: 55.2–81.4), respectively, for those with HER-low TNBC. No significant differences in survival outcomes were observed between HER2 0 and HER2-low TNBC patients in the overall NAC cohort (high-grade ST and NST combined: OS: *p* = 0.329; DDFS: *p* = 0.605; DFS: *p* = 0.287), nor within the high-grade TNBC ST (OS: *p* = 0.512; DDFS: *p* = 0.746; DFS: *p* = 0.419) or TNBC NST (OS: *p* = 0.209; DDFS: *p* = 0.496; DFS: *p* = 0.147) subgroups. HER2 status was not significantly associated with survival in regression analyses (high-grade ST: OS: HR = 0.04, *p* = 0.683; DDFS: HR = 0.70, *p* = 0.751; DFS: HR = 0.43, *p* = 0.447; S13 and S14 Tables). Within the NAC-treated cohort, patients with HER2 0 high-grade TNBC ST showed lower 5-year OS, DDFS, and DFS rates compared to those with HER2 0 TNBC NST (S11 Table). However, no significant differences in survival outcomes were found between patients with high-grade TNBC ST and those with TNBC NST within either the HER2 0 subgroup (OS: *p* = 0.590; DDFS: *p* = 0.406; DFS: *p* = 0.092) or the HER2-low subgroup (OS: *p* = 0.290; DDFS: *p* = 0.758; DFS: *p* = 0.627) (Figs 4A–D). Furthermore, TNBC subtype (high-grade ST vs. NST) was not independently associated with survival in regression analyses (S15 and S16 Tables).

## Discussion

This study provides preliminary insights into the prognostic significance of classifying TNBC into high-grade ST versus NST within HER2-low and HER2 0 expression subgroups. The findings highlight associations between HER2-low expression, clinicopathological characteristics, and survival outcomes in patients with high-grade TNBC ST compared to those with TNBC NST. Our analysis suggests that the prognostic implications of TNBC subtype (high-grade ST vs. NST) may be modulated by HER2 status in patients undergoing primary surgical treatment. In the subgroup of patients who did not receive NAC (non-NAC), those with HER2 0 high-grade TNBC ST exhibited significantly poorer survival outcomes compared to patients with HER2 0 TNBC NST. In contrast, patients with HER2-low high-grade TNBC ST who underwent primary surgery showed survival outcomes comparable to their HER2-low TNBC NST counterparts. Cox regression analysis identified high-grade TNBC ST as an independent adverse prognostic factor in the non-NAC HER2 0 subgroup, but not in the non-NAC HER2-low subgroup.

TNBC represents a heterogeneous group of malignant neoplasms, including tumors of no special type as well as various distinct histological subtypes. Based on current literature [23–29], TNBC ST can be classified into three prognostic categories: low-grade, uncertain biological potential, and high-grade. While low-grade TNBC ST generally exhibits an indolent clinical course, high-grade TNBC ST is characterized by a more aggressive biological behavior, frequently requiring systemic therapy [30]. Previous investigations examining survival outcomes in TNBC ST, without stratification by prognostic subgroup or HER2 status, have reported inferior survival in patients with triple-negative ILC [34–41], mixed-type NST-ILC [34–36], and metaplastic carcinoma [34–36] when compared to TNBC NST. A recent study [30] involving 513 patients with non-metastatic, unilateral, unifocal TNBC stratified by prognostic TNBC subgroups reported significantly worse OS, DDFS, and DFS in patients with high-grade TNBC ST compared to those with TNBC NST. These survival differences were observed across the entire cohort, including both recipients and non-recipients of NAC, as well as within the subgroup that did not receive NAC. Furthermore, multivariate regression analysis identified prognostic TNBC ST subgroups (low-grade ST, high-grade ST, and apocrine androgen receptor-positive ST/uncertain biological behavior) as independent predictors of DDFS and DFS [30]. Based on these findings, the authors underscored the importance of stratifying TNBC ST into prognostic subgroups to enhance risk assessment and optimize therapeutic strategies for patients with these rare BC entities [30].

In the present study, we investigated the prognostic significance of high-grade TNBC ST compared to TNBC NST in a cohort stratified by HER2 status. Among non-NAC patients, those with HER2 0 high-grade TNBC ST exhibited significantly poorer OS, DDFS, and DFS compared to patients with HER2 0 TNBC NST. These survival differences remained statistically significant after matching for pathological tumor size (pT category) between the non-NAC HER2 0 high-grade TNBC ST and TNBC NST subgroups. Multivariate analysis identified high-grade TNBC ST as an independent predictor of adverse survival outcomes in the non-NAC HER2 0 subgroup. In contrast, within the non-NAC HER2-low subgroup, TNBC subtype (high-grade ST vs. NST) was not independently associated with survival in either univariate or multivariate analyses. This observation underscores the necessity for further research involving larger cohorts of TNBC patients to elucidate the prognostic implications of HER2-low expression within distinct TNBC ST subgroups. In the neoadjuvant setting, no significant differences in OS, DDFS, or DFS were observed between patients with high-grade TNBC ST and those with TNBC NST, regardless of pCR status, in either the HER2 0 or HER2-low subgroups. This was observed despite the lower pCR rate among patients with high-grade TNBC ST compared to those with TNBC NST. Similarly, a recent study by Zhang et al. [42] analyzed data from the Surveillance, Epidemiology, and End Results (SEER) database and reported no significant survival differences between patients with triple-negative metaplastic BC (which accounted for 64.4% of high-grade TNBC ST cases in our cohort) and those with triple-negative invasive ductal carcinoma (TNBC-IDC) among individuals who achieved either complete or partial response following NAC. However, among patients who did not respond to neoadjuvant therapy, TNBC-IDC was associated with significantly better OS and breast cancer-specific survival (BCSS) compared to triple-negative metaplastic BC. Additionally, in patients who did not receive chemotherapy, TNBC-IDC demonstrated significantly improved OS, though not BCSS, compared to triple-negative metaplastic BC [42]. Cox regression analysis from the same study identified chemotherapy as a favorable prognostic factor in both triple-negative metaplastic BC and TNBC-IDC [42]. These findings, in conjunction with our results, suggest that patients with high-grade TNBC ST, particularly those with triple-negative metaplastic BC, may derive clinical benefit from NAC, despite exhibiting lower pCR rates compared to TNBC NST.

The prognostic significance of HER2-low status in TNBC remains a subject of ongoing debate. Prior studies have yielded inconsistent results regarding the impact of HER2-low expression on clinical outcomes in TNBC, with discrepancies likely attributable to differences in cohort sample size, follow-up duration, and selected endpoints [12]. In the present analysis, HER2-low status did not emerge as an independent prognostic factor for survival outcomes in the overall cohort (including both high-grade TNBC ST and TNBC NST cases), nor within the high-grade TNBC ST and TNBC NST subgroups. These findings align with previous studies reporting no association between HER2-low expression and survival in HER2 non-amplified, HR-negative BC across both non-neoadjuvant [7,14,15] and neoadjuvant [16–18] treatment settings. Conversely, several investigations have suggested that patients with HER2-low TNBC exhibit more favorable survival outcomes compared to those with HER2 0 TNBC [9–12]. For example, an international multicenter cohort study demonstrated significantly improved OS in patients with HR-negative, HER2-low BC compared to those with HR-negative, HER2 0 tumors [12]. Similarly, a nationwide Korean cohort study with a median follow-up of 12 years reported significantly improved BCSS in patients with HER2-low BC compared to those with HER2 0 tumors, irrespective of HR status [9]. Notably, multivariate analysis in that study identified HER2-low expression as an independent predictor of BCSS only within the TNBC subgroup [9]. In the neoadjuvant setting, a single-center Korean study observed significantly improved DFS, though not OS, in patients with non-metastatic HER2-low TNBC compared to those with HER2 0 TNBC [11]. Moreover, a pooled analysis of German neoadjuvant trials demonstrated significantly better OS and DFS in patients with HR-negative, HER2-low BC compared to those with HR-negative, HER2 0 disease, with survival differences observed exclusively in HR-negative tumors that failed to achieve pCR following NAC [10]. Notably, both the aforementioned German study [10] and other recent investigations [18,20] reported that HER2-low status was significantly associated with lower pCR rates in HR-positive BC but not in TNBC. In contrast, studies by Tarantino et al. [14] and Ilie et al. [19] found no statistically significant differences in pCR rates between non-metastatic HER2-low and HER2 0 BC, irrespective of HR status. Similarly, a recent French study reported no significant differences in pCR rates between HER2-low and HER2 0 TNBC in patients receiving NAC [16]. Consistent with these latter observations, the present study did not identify significant differences in pCR rates between HER2-low and HER2 0 tumors in the overall cohort (including high-grade ST and NST), nor within the individual high-grade TNBC ST and TNBC NST subgroups.

Previous reports have postulated that the more favorable prognosis associated with HER2-low BC may be attributable to less aggressive biological profile, including lower Ki-67 proliferation indices [9–11], and reduced histological grade [10,12,13], potentially distinct molecular characteristics underlying HER2-low IHC expression and/or differences in the intrinsic subtypes between HER2-low and HER2 0 tumors [9,22]. Using data from The Cancer Genome Atlas (TCGA), Agostinetto et al. [7] reported divergent distributions of PAM50 intrinsic subtypes between HER2 0 and HER2-low TNBC. While basal-like tumors predominated in both groups, HER2-low, HR-negative tumors exhibited a higher prevalence of HER2-enriched subtypes compared to HER2 0, HR-negative tumors [7]. In contrast to prior findings [9–11], the present study did not observe significant differences in Ki-67 proliferation indices between HER2-low and HER2 0 TNBC, neither in the overall cohort nor within the high-grade TNBC ST and TNBC NST subgroups. However, consistent with previous literature [10,12,13], significant differences in histological grade were observed between HER2-low and HER2 0 TNBC in the subgroup of patients who did not receive NAC. Nevertheless, the lower histological grade associated with HER2-low TNBC in this subgroup did not translate into improved survival outcomes.

This study has several limitations. First, as a retrospective analysis, it is inherently susceptible to selection bias. Second, owing to the rarity of the disease, the number of patients diagnosed with high-grade TNBC ST was relatively small. Additionally, as treatment standards for TNBC have evolved over time, the cohort received various adjuvant and neoadjuvant chemotherapy regimens over the 14-year study period, which may have influenced survival outcomes. It is noteworthy that HER2 status in all TNBC cases was assessed using standardized IHC according to the ASCO/CAP HER2 scoring guidelines [32,33]; however, due to the moderate inter-observer variability – particularly in distinguishing between HER2 IHC score 0 and score 1+ [43] – the reproducibility of HER2 assessment across all cases cannot be fully ensured. Finally, this study focused primarily on morphological features and HER2 IHC expression, without incorporating tumor infiltrating lymphocytes (TILs) or molecular tumor characteristics, which may also serve as important prognostic indicators.

## Conclusion

Based on the findings of our study, HER2-low expression did not emerge as an independent prognostic factor for survival in the overall cohort (comprising high-grade TNBC ST and TNBC NST), nor within the high-grade TNBC ST or TNBC NST subgroups. Among tumors treated with NAC, TNBC subtype (high-grade ST vs. NST) demonstrated no independent association with survival outcomes. In contrast, among patients who did not receive NAC, stratification of TNBC into high-grade ST and NST was independently associated with DDFS and DFS within the HER2 0 subgroup, but not within the HER2-low subgroup. These findings suggest that the prognostic value of TNBC subtyping (high-grade ST vs. NST) may be modulated by HER2 status in a subset of TNBC cases. Further research is warranted to elucidate the clinical significance of HER2-low expression across different TNBC subtypes, particularly in the context of prognostic stratification and its implications for patient outcomes.

## Supporting information

S1 Table NAC/NAI/NATT and adjuvant chemotherapy/AI/ATT regimens for high-grade TNBC ST patients with follow-up data (n = 22). (DOCX)

S2 Table NAC/NAI and adjuvant chemotherapy/AI/ATT regimens for TNBC NST patients with follow-up data (n = 164). (DOCX)

S3 Table Correlations between clinicopathological features and HER2 status in high-grade TNBC ST and TNBC NST subgroups (n = 504). (DOCX)

S4 Table Correlations between clinicopathological features and HER2 status in non-NAC high-grade TNBC ST and TNBC NST subgroups (n = 310). (DOCX)

S5 Table Correlations between clinicopathological features and TNBC subtype in non-NAC patients stratified by HER2 status. (DOCX)

S6 Table Correlations between clinicopathological features and TNBC subtype in NAC-treated patients stratified by HER2 status. (DOCX)

S7 Table Correlations between clinicopathological features and HER2 status in NAC-treated high-grade TNBC ST and TNBC NST subgroups (n = 194). (DOCX)

S8 Table Univariate and multivariate analyses of clinicopathological variables in non-NAC patients with high-grade TNBC ST and TNBC NST (n = 294). (DOCX)

S9 Table Univariate and multivariate analyses of clinicopathological variables in non-NAC patients with high-grade TNBC ST (n = 73). (DOCX)

S10 Table Univariate and multivariate analyses of clinicopathological variables in non-NAC patients with TNBC NST (n = 221). (DOCX)

S11 Table Survival outcomes in patients with high-grade TNBC ST and TNBC NST. (DOCX)

S12 Table Patient and tumor characteristics of pT-matched non-NAC HER2 0 high-grade TNBC ST and TNBC NST (n = 100). (DOCX)

S13 Table Univariate and multivariate analyses of clinicopathological variables in NAC-treated patients with high-grade TNBC ST and TNBC NST (n = 186). (DOCX)

S14 Table Univariate and multivariate analyses of clinicopathological variables in NAC-treated patients with TNBC NST (n = 164). (DOCX)

S15 Table Univariate and multivariate analyses of clinicopathological variables in NAC-treated patients with HER2 0 high-grade TNBC ST and TNBC NST (n = 125). (DOCX)

S16 Table Univariate and multivariate analyses of clinicopathological variables in NAC-treated patients with HER2 1 + /2 + high-grade TNBC ST and TNBC NST (n = 61). (DOCX)

S1 Fig Overall survival (A), distant disease-free survival (B), and disease-free survival (C) in non-NAC patients with HER2 0 high-grade TNBC ST versus those with HER2 0 TNBC NST matched for pT category. (TIF)
