# Supplementary material for: Prognostic impact of HER2-low expression in triple-negative breast cancer of high-grade special histological type and no special type
Source: PLoS One. 2025 Jun 13;20(6):e0325715. doi: 10.1371/journal.pone.0325715 (PMC12165359; doi:10.1371/journal.pone.0325715)
Supplement: S1 Table — (DOCX) [file pone.0325715.s001.docx]

**S1 Table. NAC/NAI/NATT and adjuvant chemotherapy/AI/ATT regimens for high-grade TNBC ST patients with follow-up data (n=22).**

| **No.** | **NAC/NAI/NATT** | **No. of cycles (total no. of weeks)** | **AC/AI/ATT** | **No. of cycles (total no. of weeks)** | **RCB** | **LR/DM** | **Endpoint** |
| --- | --- | --- | --- | --- | --- | --- | --- |
| 1 | AT | 4 x docetaxel (12 weeks), 4 x epirubicin (9 weeks) | No | - | II | No | Death due to other cause |
| 2 | ACT | 4 x epirubicin/cyclophosphamide (9 weeks), 4 x docetaxel (10 weeks) | Yes | 9 x capecitabine (24 weeks) | I | Yes | Death due to BC |
| 3 | ACT | 12 x Nab-paclitaxel (11 weeks), 4 x ACdd (6 weeks) | Yes | 8 x capecitabine (23 weeks) | II | No | Alive |
| 4 | ACT | 12 x Nab-paclitaxel (11 weeks), 4 x ACdd (7 weeks) | Yes | 8 x capecitabine (24 weeks) | II | No | Alive |
| 5 | ACT | 4 x epirubicin/cyclophosphamide, 12 x paclitaxel (12 weeks) | Yes | 9 x capecitabine (24 weeks) | II | No | Alive |
| 6 | AT | 6 x docetaxel/epirubicin (16 weeks) | No | - | II | No | Alive |
| 7 | TP+pembrolizumab, AC+pembrolizumab | 3 x carboplatin, 3 x pembrolizumab, 8 x paclitaxel (8 weeks due to hepatopathy), 2 x epirubicin/cyclophosphamide + 1 x pembrolizumab (6 weeks) | No | - | III | No | Alive |
| 8 | ACdd+TP | 4 x epirubicin/cyclophosphamide, 12 x paclitaxel/carboplatin (12 weeks) | Yes | 9 x capecitabine (24 weeks) | II | No | Alive |
| 9 | T+PARP inhibitor | 12 x paclitaxel (12 weeks), talazoparib (5 weeks) | Yes | PARP inhibitor (24 months) | II | Yes | Alive (stable disease) |
| 10 | PEI | cisplatin, etoposide, ifosfamide (14 weeks) | Yes | unknown | II | Yes | Alive |
| 11 | ACT | 4 x epirubicin/cyclophosphamide, 12 x paclitaxel (20 weeks) | Yes | paclitaxel (6 weeks) | II | Yes | Death due to BC |
| 12 | ATP | 18 x paclitaxel, liposomal doxorubicin, carboplatin (18 weeks) | No | - | 0 | No | Alive |
| 13 | AC+TP+capecitabine | 4 x epirubicin/cyclophosphamide, 12 x paclitaxel (14 weeks), carboplatin (12 weeks), capecitabine (8 weeks) | Yes | capecitabine (duration unknown) | II | Yes | Alive (progressive disease) |
| 14 | ATP | 12 x paclitaxel, liposomal doxorubicin, carboplatin (12 weeks) | Yes | cyclophosphamide, methotrexate, 5-fluorouracil | III | Yes | Alive (progressive disease) |
| 15 | AC | 4 x doxorubicin/cyclophosphamide (4 weeks) | Yes | 10 x carboplatin, 12 x paclitaxel (13 weeks), 8 x capecitabine (27 weeks) | III | No | Alive |
| 16 | TP+pembrolizumab, AC+pembrolizumab | 4 x carboplatin, 4 x pembrolizumab, 12 x paclitaxel (12 weeks), 4 x epirubicin/cyclophosphamide + pembrolizumab (9 weeks) | Yes | 9 x capecitabine + pembrolizumab (24 weeks) | II | No | Alive |
| 17 | T | 6 x paclitaxel (6 weeks) | Yes | 4 x ACdd (6 weeks) | II | No | Alive |
| 18 | ACT | 3 x epirubicin/cyclophosphamide, 12 x paclitaxel (12 weeks) | No | - | 0 | No | Alive |
| 19 | TP+pembrolizumab, AC+pembrolizumab | 4 x carboplatin, 4 x pembrolizumab, 12 x paclitaxel (12 weeks), 4 x epirubicin/cyclophosphamide + pembrolizumab (9 weeks) | Yes | 9 x capecitabine + pembrolizumab (24 weeks) | II | No | Alive |
| 20 | ACT | 4 x epirubicin/cyclophosphamide, 12 x paclitaxel (19 weeks) | Yes | 8 x capecitabine (23 weeks) | II | No | Alive |
| 21 | AC+TP | 4 x doxorubicin/cyclophosphamide, 4 x carboplatin, 12 x paclitaxel (14 weeks) | Yes | 9 x capecitabine (24 weeks) | II | No | Alive |
| 22 | ACdd+TP | 4 x epirubicin/cyclophosphamide, 4 x carboplatin, 12 x paclitaxel (12 weeks) | No | - | I | No | Alive |

NAC neoadjuvant chemotherapy, NAI neoadjuvant immunotherapy, NATT neoadjuvant targeted therapy, AC adjuvant chemotherapy, AI adjuvant immunotherapy, ATT adjuvant targeted therapy, TNBC ST triple-negative breast cancer special type, A anthracycline, C cyclophosphamide, T taxane, P platinum, BC breast cancer.
