# Supplementary material for: Prognostic impact of HER2-low expression in triple-negative breast cancer of high-grade special histological type and no special type
Source: PLoS One. 2025 Jun 13;20(6):e0325715. doi: 10.1371/journal.pone.0325715 (PMC12165359; doi:10.1371/journal.pone.0325715)
Supplement: S2 Table — (DOCX) [file pone.0325715.s002.docx]

**S2 Table. NAC/NAI and adjuvant chemotherapy/AI/ATT regimens for TNBC NST patients with follow-up data (n=164).**

| **NAC/NAI** | **N (%)** |
| --- | --- |
| ACT | 128 (78.0) |
| without additional agents | 84 (51.2) |
| +P (carboplatin)+pembrolizumab | 23 (14.0) |
| +P (carboplatin) | 19 (11.6) |
| +5-fluorouracil | 2 (1.2) |
| AT | 23 (14.0) |
| without additional agents | 18 (11.0) |
| +capecitabine | 3 (1.8) |
| +carboplatin | 2 (1.2) |
| TP (carboplatin) | 5 (3.0) |
| CT | 4 (2.4) |
| without additional agents | 3 (1.8) |
| +P (cisplatin) | 1 (0.6) |
| AC | 3 (1.8) |
| P (carboplatin)+gemcitabine | 1 (0.6) |
| **Adjuvant chemotherapy** | **N (%)** |
| capecitabine | 34 (20.7) |
| without additional agents | 29 (17.7) |
| +pembrolizumab | 4 (2.4) |
| +ACT/P (carboplatin) | 1 (0.6) |
| P (carboplatin) | 5 (3.0) |
| AC | 3 (1.8) |
| T (docetaxel/paclitaxel) | 2 (1.2) |
| **AIT/ATT** | **N (%)** |
| pembrolizumab | 22 (13.4) |
| without additional agents | 17 (10.4) |
| +capecitabine | 4 (2.4) |
| +PARP inhibitor | 1 (0.6) |
| PARP inhibitor (without additional agents) | 3 (1.8) |
| sacituzumab-govitecan | 1 (0.6) |

NAC neoadjuvant chemotherapy, NAI neoadjuvant immunotherapy, AI adjuvant immunotherapy, ATT adjuvant targeted therapy, TNBC NST triple-negative breast cancer of no special type, A anthracycline, C cyclophosphamide, T taxane, P platinum.
