# Supplementary material for: Prognostic impact of HER2-low expression in triple-negative breast cancer of high-grade special histological type and no special type
Source: PLoS One. 2025 Jun 13;20(6):e0325715. doi: 10.1371/journal.pone.0325715 (PMC12165359; doi:10.1371/journal.pone.0325715)
Supplement: S3 Table — (DOCX) [file pone.0325715.s003.docx]

**S3 Table.** **Correlations between clinicopathological features and HER2 status in high-grade TNBC ST and TNBC NST subgroups (n=504).**

|  | **Overall (n=504)** | | | | **TNBC ST high-grade (n=104)** | | | | **TNBC NST (n=400)** | | | | |
| --- | --- | --- | --- | --- | --- | --- | --- | --- | --- | --- | --- | --- | --- |
| **Variable** | **HER2 0 (n=333)** | **HER2 1+/2+ (n=171)** | |  | **HER2 0**  **(n=73)** | **HER2 1+/2+ (n=31)** | |  | | **HER2 0 (n=260)** | **HER2 1+/2+ (n=140)** | |  |
|  | **N (%)** | **N (%)** | ***p*-Value** | | **N (%)** | **N (%)** | ***p*-Value** | | | **N (%)** | **N (%)** | ***p*-Value** | |
| **Age group** (years) |  |  |  | |  |  |  | | |  |  |  | |
| < 50 | 149 (44.7) | 65 (38.0) | 0.148 | | 23 (31.5) | 9 (29.0) | 0.803 | | | 126 (48.5) | 56 (40.0) | 0.105 | |
| ≥ 50 | 184 (55.3) | 106 (62.0) |  | | 50 (68.5) | 22 (71.0) |  | | | 134 (51.5) | 84 (60.0) |  | |
| **Mean age** (years) | 53.8±15.8 | 56.7±15.1 | **0.048** | | 57.6±17.3 | 59.8±12.1 | 0.513 | | | 52.7±15.2 | 56.0±15.6 | **0.042** | |
| **Year of diagnosis** |  |  |  | |  |  |  | | |  |  |  | |
| 2010-2017 | 201 (60.4) | 90 (52.6) | 0.096 | | 45 (61.6) | 17 (54.8) | 0.518 | | | 156 (60.0) | 73 (52.1) | 0.130 | |
| 2018-2023 | 132 (39.6) | 81 (47.4) |  | | 28 (38.4) | 14 (45.2) |  | | | 104 (40.0) | 67 (47.9) |  | |
| **c/pT category** |  |  |  | |  |  |  | | |  |  |  | |
| T1 | 137 (41.1) | 76 (44.4) | 0.406 | | 24 (32.9) | 11 (35.5) | 0.393 | | | 113 (43.5) | 65 (46.4) | 0.778 | |
| T2 | 147 (44.1) | 77 (45.0) |  | | 31 (42.5) | 16 (51.6) |  | | | 116 (44.6) | 61 (43.6) |  | |
| T3/T4 | 49 (14.7) | 18 (10.5) |  | | 18 (24.7) | 4 (12.9) |  | | | 31 (11.9) | 14 (10.0) |  | |
| **c/pN category** |  |  |  | |  |  |  | | |  |  |  | |
| N0 | 205 (61.6) | 119 (69.6) | 0.189 | | 47 (64.4) | 26 (83.9) | 0.099 | | | 158 (60.8) | 93 (66.4) | 0.536 | |
| N1/N1mi | 95 (28.5) | 37 (21.6) |  | | 21 (28.8) | 3 (9.7) |  | | | 74 (28.5) | 34 (24.3) |  | |
| N2/N3 | 33 (9.9) | 15 (8.8) |  | | 5 (6.8) | 2 (6.5) |  | | | 28 (10.8) | 13 (9.3) |  | |
| **Nodal status** (post-NAC) |  |  |  | |  |  |  | | |  |  |  | |
| N- | 246 (73.9) | 132 (77.2) | 0.415 | | 51 (69.9) | 26 (83.9) | 0.136 | | | 195 (75.0) | 106 (75.7) | 0.875 | |
| N+ | 87 (26.1) | 39 (22.8) |  | | 22 (30.1) | 5 (16.1) |  | | | 65 (25.0) | 34 (24.3) |  | |
| **Ki-67 index** (%) |  |  |  | |  |  |  | | |  |  |  | |
| ≤ 20 | 14 (4.2) | 10 (5.8) | 0.412 | | 9 (12.3) | 6 (19.4) | 0.351 | | | 5 (1.9) | 4 (2.9) | 0.548 | |
| > 20 | 319 (95.8) | 161 (94.2) |  | | 64 (87.7) | 25 (80.6) |  | | | 255 (98.1) | 136 (97.1) |  | |
| **Mean Ki-67 index** (%) | 59.9±21.8 | 61.3±21.8 | 0.470 | | 53.0±24.0 | 49.1±24.1 | 0.456 | | | 61.8±20.8 | 64.0±20.4 | 0.298 | |
| **Grade*** |  |  |  | |  |  |  | | |  |  |  | |
| G2 | 25 (7.7) | 20 (12.0) | 0.119 | | 6 (9.2) | 7 (25.9) | **0.036** | | | 19 (7.3) | 13 (9.3) | 0.487 | |
| G3 | 300 (92.3) | 147 (88.0) |  | | 59 (90.8) | 20 (74.1) |  | | | 241 (92.7) | 127 (90.7) |  | |
| **NAC** |  |  |  | |  |  |  | | |  |  |  | |
| Yes | 129 (38.7) | 65 (38.0) | 0.874 | | 16 (21.9) | 8 (25.8) | 0.667 | | | 113 (43.5) | 57 (40.7) | 0.596 | |
| No | 204 (61.3) | 106 (62.0) |  | | 57 (78.1) | 23 (74.2) |  | | | 147 (56.5) | 83 (59.3) |  | |
| **Surgery type** |  |  |  | |  |  |  | | |  |  |  | |
| BCT | 208 (62.5) | 106 (62.0) | 0.917 | | 36 (49.3) | 20 (64.5) | 0.155 | | | 172 (66.2) | 86 (61.4) | 0.346 | |
| Mastectomy | 125 (37.5) | 65 (38.0) |  | | 37 (50.7) | 11 (35.5) |  | | | 88 (33.8) | 54 (38.6) |  | |
| **Adjuvant CT** (missing: 22) |  |  |  | |  |  |  | | |  |  |  | |
| Yes | 195 (61.3) | 101 (61.6) | 0.955 | | 49 (74.2) | 23 (74.2) | 0.996 | | | 146 (57.9) | 78 (58.6) | 0.893 | |
| No | 123 (38.7) | 63 (38.4) |  | | 17 (25.8) | 8 (25.8) |  | | | 106 (42.1) | 55 (41.4) |  | |
| **Adjuvant RT** (missing: 22) |  |  |  | |  |  |  | | |  |  |  | |
| Yes | 257 (80.8) | 116 (70.7) | **0.012** | | 54 (81.8) | 20 (64.5) | 0.062 | | | 203 (80.6) | 96 (72.2) | 0.061 | |
| No | 61 (19.2) | 48 (29.3) |  | | 12 (18.2) | 11 (35.5) |  | | | 49 (19.4) | 37 (27.8) |  | |

TNBC triple-negative breast cancer, ST special type, NST no special type, NAC neoadjuvant chemotherapy, BCT breast conserving therapy, CT chemotherapy, RT radiotherapy. *No grading according to WHO 2019 in adenoid-cystic carcinoma.
