# Supplementary material for: Prognostic impact of HER2-low expression in triple-negative breast cancer of high-grade special histological type and no special type
Source: PLoS One. 2025 Jun 13;20(6):e0325715. doi: 10.1371/journal.pone.0325715 (PMC12165359; doi:10.1371/journal.pone.0325715)
Supplement: S4 Table — (DOCX) [file pone.0325715.s004.docx]

**S4 Table.** **Correlations between clinicopathological features and HER2 status in non-NAC high-grade TNBC ST and TNBC NST subgroups (n=310).**

|  | **Overall (n=310)** | | | | **TNBC ST high-grade (n=80)** | | | | **TNBC NST (n=230)** | | | | |
| --- | --- | --- | --- | --- | --- | --- | --- | --- | --- | --- | --- | --- | --- |
| **Variable** | **HER2 0 (n=204)** | **HER2 1+/2+ (n=106)** | |  | **HER2 0 (n=57)** | **HER2 1+/2+ (n=23)** | |  | | **HER2 0 (n=147)** | **HER2 1+/2+ (n=83)** | |  |
|  | **N (%)** | **N (%)** | ***p*-Value** | | **N (%)** | **N (%)** | ***p*-Value** | | | **N (%)** | **N (%)** | ***p*-Value** | |
| **Age group** (years) |  |  |  | |  |  |  | | |  |  |  | |
| < 50 | 61 (29.9) | 30 (28.3) | 0.769 | | 12 (21.1) | 6 (26.1) | 0.626 | | | 49 (33.3) | 24 (28.9) | 0.489 | |
| ≥ 50 | 143 (70.1) | 76 (71.7) |  | | 45 (78.9) | 17 (73.9) |  | | | 98 (66.7) | 59 (71.1) |  | |
| **Mean age** (years) | 59.1±16.0 | 60.4±15.0 | 0.494 | | 62.1±16.0 | 60.0±12.1 | 0.575 | | | 58.0±15.8 | 60.5±15.8 | 0.242 | |
| **Year of diagnosis** |  |  |  | |  |  |  | | |  |  |  | |
| 2010-2017 | 135 (66.2) | 67 (63.2) | 0.603 | | 34 (59.6) | 17 (73.9) | 0.230 | | | 101 (68.7) | 50 (60.2) | 0.194 | |
| 2018-2023 | 69 (33.8) | 39 (36.8) |  | | 23 (40.4) | 6 (26.1) |  | | | 46 (31.3) | 33 (39.8) |  | |
| **pT category** |  |  |  | |  |  |  | | |  |  |  | |
| T1 | 99 (48.5) | 53 (50.0) | 0.124 | | 21 (36.8) | 9 (39.1) | 0.601 | | | 78 (53.1) | 44 (53.0) | 0.251 | |
| T2 | 76 (37.3) | 46 (43.4) |  | | 23 (40.4) | 11 (47.8) |  | | | 53 (36.1) | 35 (42.2) |  | |
| T3/T4 | 29 (14.2) | 7 (6.6) |  | | 13 (22.8) | 3 (13.0) |  | | | 16 (10.9) | 4 (4.8) |  | |
| **pN category** |  |  |  | |  |  |  | | |  |  |  | |
| N0 | 143 (70.1) | 83 (78.3) | 0.278 | | 40 (70.2) | 19 (82.6) | 0.257 | | | 103 (70.1) | 64 (77.1) | 0.268 | |
| N1/N1mi | 42 (20.6) | 17 (16.0) |  | | 14 (24.6) | 2 (8.7) |  | | | 28 (19.0) | 15 (18.1) |  | |
| pN2/N3 | 19 (9.3) | 6 (5.7) |  | | 3 (5.3) | 2 (8.7) |  | | | 16 (10.9) | 4 (4.8) |  | |
| **Nodal status** |  |  |  | |  |  |  | | |  |  |  | |
| N- | 143 (70.1) | 83 (78.3) | 0.123 | | 40 (70.2) | 19 (82.6) | 0.253 | | | 103 (70.1) | 64 (77.1) | 0.250 | |
| N+ | 61 (29.9) | 23 (21.7) |  | | 17 (29.8) | 4 (17.4) |  | | | 44 (29.9) | 19 (22.9) |  | |
| **Mean tumor size** (mm) | 29.0±25.5 | 24.3±14.2 | **0.035** | | 37.3±33.5 | 27.4±16.5 | 0.084 | | | 25.8±20.8 | 23.4±13.5 | 0.280 | |
| **Ki-67 index** (%) |  |  |  | |  |  |  | | |  |  |  | |
| **≤ 20** | 10 (4.9) | 9 (8.5) | 0.211 | | 8 (14.0) | 5 (21.7) | 0.398 | | | 2 (1.4) | 4 (4.8) | 0.114 | |
| **> 20** | 194 (95.1) | 97 (91.5) |  | | 49 (86.0) | 18 (78.3) |  | | | 145 (98.6) | 79 (95.2) |  | |
| **Mean Ki-67 index** (%) | 57.8±22.6 | 59.1±23.0 | 0.638 | | 51.7±25.3 | 49.0±24.9 | 0.668 | | | 60.2±21.0 | 61.9±21.8 | 0.560 | |
| **Grade*** |  |  |  | |  |  |  | | |  |  |  | |
| G2 | 10 (5.1) | 13 (12.7) | **0.019** | | 5 (10.2) | 5 (26.3) | 0.092 | | | 5 (3.4) | 8 (9.6) | **0.049** | |
| G3 | 186 (94.9) | 89 (87.3) |  | | 44 (89.8) | 14 (73.7) |  | | | 142 (96.6) | 75 (90.4) |  | |
| **Surgery type** |  |  |  | |  |  |  | | |  |  |  | |
| BCT | 130 (63.7) | 70 (66.0) | 0.686 | | 30 (52.6) | 14 (60.9) | 0.503 | | | 100 (68.0) | 56 (67.5) | 0.931 | |
| Mastectomy | 74 (36.3) | 36 (34.0) |  | | 27 (47.4) | 9 (39.1) |  | | | 47 (32.0) | 27 (32.5) |  | |
| **Adjuvant CT** (missing: 15) |  |  |  | |  |  |  | | |  |  |  | |
| Yes | 154 (80.2) | 82 (79.6) | 0.903 | | 39 (76.5) | 17 (73.9) | 0.812 | | | 115 (81.6) | 65 (81.3) | 0.955 | |
| No | 38 (19.8) | 21 (20.4) |  | | 12 (23.5) | 6 (26.1) |  | | | 26 (18.4) | 15 (18.8) |  | |
| **Adjuvant RT** (missing: 15) |  |  |  | |  |  |  | | |  |  |  | |
| Yes | 152 (79.2) | 68 (66.0) | **0.013** | | 42 (82.4) | 12 (52.2) | **0.007** | | | 110 (78.0) | 56 (70.0) | 0.185 | |
| No | 40 (20.8) | 35 (34.0) |  | | 9 (17.6) | 11 (47.8) |  | | | 31 (22.0) | 24 (30.0) |  | |

TNBC triple-negative breast cancer, ST special type, NST no special type, BCT breast conserving therapy, CT chemotherapy, RT radiotherapy. *No grading according to WHO 2019 in adenoid-cystic carcinoma.
