# Supplementary material for: Prognostic impact of HER2-low expression in triple-negative breast cancer of high-grade special histological type and no special type
Source: PLoS One. 2025 Jun 13;20(6):e0325715. doi: 10.1371/journal.pone.0325715 (PMC12165359; doi:10.1371/journal.pone.0325715)
Supplement: S5 Table — (DOCX) [file pone.0325715.s005.docx]

**S5 Table.** **Correlations between clinicopathological features and TNBC subtype in non-NAC patients stratified by HER2 status.**

|  | **HER2 0 TNBC** | | | | **HER2 1+/2+ TNBC** | | | | |
| --- | --- | --- | --- | --- | --- | --- | --- | --- | --- |
| **Variable** | **Overall (n=204)** | **NST (n=147)** | **ST high-grade (n=57)** |  | | **Overall (n=106)** | **NST (n=83)** | **ST high-grade (n=23)** |  |
|  | **N (%)** | **N (%)** | **N (%)** | ***p*-Value** | | **N (%)** | **N (%)** | **N (%)** | ***p*-Value** |
| **Age group** (years) |  |  |  |  | |  |  |  |  |
| < 50 | 61 (29.9) | 49 (33.3) | 12 (21.1) | 0.086 | | 30 (28.3) | 24 (28.9) | 6 (26.1) | 0.790 |
| ≥ 50 | 143 (70.1) | 98 (66.7) | 45 (78.9) |  | | 76 (71.7) | 59 (71.1) | 17 (73.9) |  |
| **Mean age** (years) | 59.1±16.0 | 58.0±15.8 | 62.1±16.0 | 0.097 | | 60.4±15.0 | 60.5±15.8 | 60.0±12.1 | 0.887 |
| **Year of diagnosis** |  |  |  |  | |  |  |  |  |
| 2010-2017 | 135 (66.2) | 101 (68.7) | 34 (59.6) | 0.220 | | 67 (63.2) | 50 (60.2) | 17 (73.9) | 0.229 |
| 2018-2023 | 69 (33.8) | 46 (31.3) | 23 (40.4) |  | | 39 (36.8) | 33 (39.8) | 6 (26.1) |  |
| **pT category** |  |  |  |  | |  |  |  |  |
| T1 | 99 (48.5) | 78 (53.1) | 21 (36.8) | **0.038** | | 53 (50.0) | 44 (53.0) | 9 (39.1) | 0.263 |
| T2 | 76 (37.3) | 53 (36.1) | 23 (40.4) |  | | 46 (43.4) | 35 (42.2) | 11 (47.8) |  |
| T3/T4 | 29 (14.2) | 16 (10.9) | 13 (22.8) |  | | 7 (6.6) | 4 (4.8) | 3 (13.0) |  |
| **pN category** |  |  |  |  | |  |  |  |  |
| N0 | 143 (70.1) | 103 (70.1) | 40 (70.2) | 0.368 | | 83 (78.3) | 64 (77.1) | 19 (82.6) | 0.464 |
| N1/N1mi | 42 (20.6) | 28 (19.0) | 14 (24.6) |  | | 17 (16.0) | 15 (18.1) | 2 (8.7) |  |
| N2/N3 | 19 (9.3) | 16 (10.9) | 3 (5.3) |  | | 6 (5.7) | 4 (4.8) | 2 (8.7) |  |
| **Nodal status** |  |  |  |  | |  |  |  |  |
| N- | 143 (70.1) | 103 (70.1) | 40 (70.2) | 0.988 | | 83 (78.3) | 64 (77.1) | 19 (82.6) | 0.571 |
| N+ | 61 (29.9) | 44 (29.9) | 17 (29.8) |  | | 23 (21.7) | 19 (22.9) | 4 (17.4) |  |
| **Ki-67 index** (%) |  |  |  |  | |  |  |  |  |
| ≤ 20 | 10 (4.9) | 2 (1.4) | 8 (14.0) | **<0.001** | | 9 (8.5) | 4 (4.8) | 5 (21.7) | **0.010** |
| > 20 | 194 (95.1) | 145 (98.6) | 49 (86.0) |  | | 97 (91.5) | 79 (95.2) | 18 (78.3) |  |
| **Mean Ki-67 index** (%) | 57.8±22.6 | 60.2±21.0 | 51.7±25.3 | **0.028** | | 59.1±23.0 | 61.9±21.8 | 49.0±24.9 | **0.017** |
| **Grade*** |  |  |  |  | |  |  |  |  |
| G2 | 10 (5.1) | 5 (3.4) | 5 (10.2) | 0.061 | | 13 (12.7) | 8 (9.6) | 5 (26.3) | **0.049** |
| G3 | 186 (94.9) | 142 (96.6) | 44 (89.8) |  | | 89 (87.3) | 75 (90.4) | 14 (73.7) |  |
| **Surgery type** |  |  |  |  | |  |  |  |  |
| BCT | 130 (63.7) | 100 (68.0) | 30 (52.6) | **0.040** | | 70 (66.0) | 56 (67.5) | 14 (60.9) | 0.554 |
| Mastectomy | 74 (36.3) | 47 (32.0) | 27 (47.4) |  | | 36 (34.0) | 27 (32.5) | 9 (39.1) |  |
| **Adjuvant CT** (missing: 15) |  |  |  |  | |  |  |  |  |
| Yes | 154 (80.2) | 115 (81.6) | 39 (76.5) | 0.434 | | 82 (79.6) | 65 (81.3) | 17 (73.9) | 0.441 |
| No | 38 (19.8) | 26 (18.4) | 12 (23.5) |  | | 21 (20.4) | 15 (18.8) | 6 (26.1) |  |
| **Adjuvant RT** (missing: 15) |  |  |  |  | |  |  |  |  |
| Yes | 152 (79.2) | 110 (78.0) | 42 (82.4) | 0.513 | | 68 (66.0) | 56 (70.0) | 12 (52.2) | 0.112 |
| No | 40 (20.8) | 31 (22.0) | 9 (17.6) |  | | 35 (34.0) | 24 (30.0) | 11 (47.8) |  |
| **Adjuvant IT** (missing: 15) |  |  |  |  | |  |  |  |  |
| Yes | 5 (2.6) | 5 (3.5) | 0 (0.0) | 0.173 | | 2 (1.9) | 1 (1.3) | 1 (4.3) | 0.343 |
| No | 187 (97.4) | 136 (96.5) | 51 (100.0) |  | | 101 (98.1) | 79 (98.8) | 22 (95.7) |  |

TNBC triple-negative breast cancer, ST special type, NST no special type, BCT breast conserving therapy, CT chemotherapy, RT radiotherapy, IT immunotherapy. *No grading according to WHO 2019 in adenoid-cystic carcinoma.
