# Supplementary material for: Prognostic impact of HER2-low expression in triple-negative breast cancer of high-grade special histological type and no special type
Source: PLoS One. 2025 Jun 13;20(6):e0325715. doi: 10.1371/journal.pone.0325715 (PMC12165359; doi:10.1371/journal.pone.0325715)
Supplement: S6 Table — (DOCX) [file pone.0325715.s006.docx]

**S6 Table. Correlations between clinicopathological features and TNBC subtype in NAC-treated patients stratified by HER2 status.**

|  | **HER2 0 TNBC** | | | | **HER2 1+/2+ TNBC** | | | | |
| --- | --- | --- | --- | --- | --- | --- | --- | --- | --- |
| **Variable** | **Overall (n=129)** | **NST (n=113)** | **ST high-grade (n=16)** |  | | **Overall (n=65)** | **NST (n=57)** | **ST high-grade (n=8)** |  |
|  | **N (%)** | **N (%)** | **N (%)** | ***p*-Value** | | **N (%)** | **N (%)** | **N (%)** | ***p*-Value** |
| **Age group** (years) |  |  |  |  | |  |  |  |  |
| < 50 | 88 (68.2) | 77 (68.1) | 11 (68.8) | 0.961 | | 35 (53.8) | 32 (56.1) | 3 (37.5) | 0.322 |
| ≥ 50 | 41 (31.8) | 36 (31.9) | 5 (31.3) |  | | 30 (46.2) | 25 (43.9) | 5 (62.5) |  |
| **Mean age** (years) | 45.4±11.1 | 45.9±11.0 | 41.6±11.6 | 0.146 | | 50.7±13.2 | 49.4±12.8 | 59.4±12.9 | **0.044** |
| **Year of diagnosis** |  |  |  |  | |  |  |  |  |
| 2010-2017 | 66 (51.2) | 55 (48.7) | 11 (68.8) | 0.133 | | 23 (35.4) | 23 (40.4) | 0 (0.0) | **0.025** |
| 2018-2023 | 63 (48.8) | 58 (51.3) | 5 (31.3) |  | | 42 (64.6) | 34 (59.6) | 8 (100.0) |  |
| **ypT category** |  |  |  |  | |  |  |  |  |
| T0 | 67 (51.9) | 65 (57.5) | 2 (12.5) | **0.001** | | 27 (41.5) | 26 (45.6) | 1 (12.5) | 0.203 |
| T1 | 38 (29.5) | 29 (25.7) | 9 (56.3) |  | | 27 (41.5) | 21 (36.8) | 6 (75.0) |  |
| T2 | 17 (13.2) | 15 (13.3) | 2 (12.5) |  | | 2 (3.1) | 2 (3.5) | 0 (0.0) |  |
| T3/T4 | 7 (5.4) | 4 (3.5) | 3 (18.8) |  | | 9 (13.8) | 8 (14.0) | 1 (12.5) |  |
| **ypN category** |  |  |  |  | |  |  |  |  |
| N0 | 103 (79.8) | 92 (81.4) | 11 (68.8) | 0.141 | | 49 (75.4) | 42 (73.7) | 7 (87.5) | 0.480 |
| N1/N1mi | 17 (13.2) | 15 (13.3) | 2 (12.5) |  | | 7 (10.8) | 6 (10.5) | 1 (12.5) |  |
| N2/N3 | 9 (7.0) | 6 (5.3) | 3 (18.8) |  | | 9 (13.8) | 9 (15.8) | 0 (0.0) |  |
| **cT stage** |  |  |  |  | |  |  |  |  |
| T1 | 38 (29.5) | 35 (31.0) | 3 (18.8) | 0.156 | | 23 (35.4) | 21 (36.8) | 2 (25.0) | 0.669 |
| T2 | 71 (55.0) | 63 (55.8) | 8 (50.0) |  | | 31 (47.7) | 26 (45.6) | 5 (62.5) |  |
| T3/T4 | 20 (15.5) | 15 (13.3) | 5 (31.3) |  | | 11 (16.9) | 10 (17.5) | 1 (12.5) |  |
| **cN stage** |  |  |  |  | |  |  |  |  |
| N0 | 62 (48.1) | 55 (48.7) | 7 (43.8) | 0.929 | | 36 (55.4) | 29 (50.9) | 7 (87.5) | 0.139 |
| N1 | 53 (41.1) | 46 (40.7) | 7 (43.8) |  | | 20 (30.8) | 19 (33.3) | 1 (12.5) |  |
| N2/N3 | 14 (10.9) | 12 (10.6) | 2 (12.5) |  | | 9 (13.8) | 9 (15.8) | 0 (0.0) |  |
| **Ki-67 index** (%) |  |  |  |  | |  |  |  |  |
| ≤ 20 | 4 (3.1) | 3 (2.7) | 1 (6.3) | 0.437 | | 1 (1.5) | 0 (0.0) | 1 (12.5) | **0.007** |
| > 20 | 125 (96.9) | 110 (97.3) | 15 (93.8) |  | | 64 (98.5) | 57 (100.0) | 7 (87.5) |  |
| **Mean Ki-67 index** (%) | 63.1±20.3 | 63.9±20.5 | 57.5±18.7 | 0.240 | | 65.0±19.4 | 67.2±18.0 | 49.4±23.2 | **0.014** |
| **Grade** |  |  |  |  | |  |  |  |  |
| G2 | 15 (11.6) | 14 (12.4) | 1 (6.3) | 0.473 | | 7 (10.8) | 5 (8.8) | 2 (25.0) | 0.166 |
| G3 | 114 (88.4) | 99 (87.6) | 15 (93.8) |  | | 58 (89.2) | 52 (91.2) | 6 (75.0) |  |
| **pCR** |  |  |  |  | |  |  |  |  |
| Yes | 63 (48.8) | 61 (54.0) | 2 (12.5) | **0.002** | | 25 (38.5) | 25 (43.9) | 0 (0.0) | **0.017** |
| No | 66 (51.2) | 52 (46.0) | 14 (87.5) |  | | 40 (61.5) | 32 (56.1) | 8 (100.0) |  |
| **Mean RCB score** | 1.207±1.425 | 1.048±1.377 | 2.356±1.258 | **<0.001** | | 1.429±1.497 | 1.333±1.572 | 2.078±0.502 | **0.011** |
| **NAI** (missing: 7) |  |  |  |  | |  |  |  |  |
| Yes | 15 (11.9) | 15 (13.5) | 0 (0.0) | 0.129 | | 12 (19.7) | 8 (15.1) | 4 (50.0) | **0.021** |
| No | 111 (88.1) | 96 (86.5) | 15 (100.0) |  | | 49 (80.3) | 45 (84.9) | 4 (50.0) |  |
| **Surgery type** |  |  |  |  | |  |  |  |  |
| BCT | 78 (60.5) | 72 (63.7) | 6 (37.5) | **0.045** | | 36 (55.4) | 30 (52.6) | 6 (75.0) | 0.233 |
| Mastectomy | 51 (39.5) | 41 (36.3) | 10 (62.5) |  | | 29 (44.6) | 27 (47.4) | 2 (25.0) |  |
| **Adjuvant CT** (missing: 7) |  |  |  |  | |  |  |  |  |
| Yes | 41 (32.5) | 31 (27.9) | 10 (66.7) | **0.003** | | 19 (31.1) | 13 (24.5) | 6 (75.0) | **0.004** |
| No | 85 (67.5) | 80 (72.1) | 5 (33.3) |  | | 42 (68.9) | 40 (75.5) | 2 (25.0) |  |
| **Adjuvant RT** (missing: 7) |  |  |  |  | |  |  |  |  |
| Yes | 105 (83.3) | 93 (83.8) | 12 (80.0) | 0.712 | | 48 (78.7) | 40 (75.5) | 8 (100.0) | 0.114 |
| No | 21 (16.7) | 18 (16.2) | 3 (20.0) |  | | 13 (21.3) | 13 (24.5) | 0 (0.0) |  |
| **Adjuvant IT** (missing: 7) |  |  |  |  | |  |  |  |  |
| Yes | 18 (14.3) | 17 (15.3) | 1 (6.7) | 0.369 | | 11 (18.0) | 9 (17.0) | 2 (25.0) | 0.582 |
| No | 108 (85.7) | 94 (84.7) | 14 (93.3) |  | | 50 (82.0) | 44 (83.0) | 6 (75.0) |  |

TNBC triple-negative breast cancer, ST special type, NST no special type, NAC neoadjuvant chemotherapy, pCR pathological complete response, RCB residual cancer burden, NAI neoadjuvant immunotherapy, BCT breast conserving therapy, CT chemotherapy, RT radiotherapy, IT immunotherapy.
