# Supplementary material for: Prognostic impact of HER2-low expression in triple-negative breast cancer of high-grade special histological type and no special type
Source: PLoS One. 2025 Jun 13;20(6):e0325715. doi: 10.1371/journal.pone.0325715 (PMC12165359; doi:10.1371/journal.pone.0325715)
Supplement: S7 Table — (DOCX) [file pone.0325715.s007.docx]

**S7 Table.** **Correlations between clinicopathological features and HER2 status in NAC-treated high-grade TNBC ST and TNBC NST subgroups (n=194).**

|  | **Overall (n=194)** | | | | **TNBC ST high-grade (n=24)** | | | | **TNBC NST (n=170)** | | | | |
| --- | --- | --- | --- | --- | --- | --- | --- | --- | --- | --- | --- | --- | --- |
| **Variable** | **HER2 0 (n=129)** | **HER2 1+/2+ (n=65)** | |  | **HER2 0 (n=16)** | **HER2 1+/2+ (n=8)** | |  | | **HER2 0 (n=113)** | **HER2 1+/2+ (n=57)** | |  |
|  | **N (%)** | **N (%)** | ***p*-Value** | | **N (%)** | **N (%)** | ***p*-Value** | | | **N (%)** | **N (%)** | ***p*-Value** | |
| **Age group** (years) |  |  |  | |  |  |  | | |  |  |  | |
| < 50 | 88 (68.2) | 35 (53.8) | 0.050 | | 11 (68.8) | 3 (37.5) | 0.143 | | | 77 (68.1) | 32 (56.1) | 0.124 | |
| ≥ 50 | 41 (31.8) | 30 (46.2) |  | | 5 (31.3) | 5 (62.5) |  | | | 36 (31.9) | 25 (43.9) |  | |
| **Mean age** (years) | 45.4±11.1 | 50.7±13.2 | **0.004** | | 41.6±11.6 | 59.4±12.9 | **0.002** | | | 45.9±11.0 | 49.4±12.8 | 0.065 | |
| **Year of diagnosis** |  |  |  | |  |  |  | | |  |  |  | |
| 2010-2017 | 66 (51.2) | 23 (35.4) | **0.037** | | 11 (68.8) | 0 (0.0) | **0.001** | | | 55 (48.7) | 23 (40.4) | 0.304 | |
| 2018-2023 | 63 (48.8) | 42 (64.6) |  | | 5 (31.3) | 8 (100.0) |  | | | 58 (51.3) | 34 (59.6) |  | |
| **ypT category** |  |  |  | |  |  |  | | |  |  |  | |
| T0 | 67 (51.9) | 27 (41.5) | **0.011** | | 2 (12.5) | 1 (12.5) | 0.700 | | | 65 (57.5) | 26 (45.6) | **0.007** | |
| T1 | 38 (29.5) | 27 (41.5) |  | | 9 (56.3) | 6 (75.0) |  | | | 29 (25.7) | 21 (36.8) |  | |
| T2 | 17 (13.2) | 2 (3.1) |  | | 2 (12.5) | 0 (0.0) |  | | | 15 (13.3) | 2 (3.5) |  | |
| T3/T4 | 7 (5.4) | 9 (13.8) |  | | 3 (18.8) | 1 (12.5) |  | | | 4 (3.5) | 8 (14.0) |  | |
| **ypN category** |  |  |  | |  |  |  | | |  |  |  | |
| N0 | 103 (79.8) | 49 (75.4) | 0.285 | | 11 (68.8) | 7 (87.5) | 0.417 | | | 92 (81.4) | 42 (73.7) | 0.073 | |
| N1/N1mi | 17 (13.2) | 7 (10.8) |  | | 2 (12.5) | 1 (12.5) |  | | | 15 (13.3) | 6 (10.5) |  | |
| N2/N3 | 9 (7.0) | 9 (13.8) |  | | 3 (18.8) | 0 (0.0) |  | | | 6 (5.3) | 9 (15.8) |  | |
| **cT stage** |  |  |  | |  |  |  | | |  |  |  | |
| T1 | 38 (29.5) | 23 (35.4) | 0.612 | | 3 (18.8) | 2 (25.0) | 0.605 | | | 35 (31.0) | 21 (36.8) | 0.447 | |
| T2 | 71 (55.0) | 31 (47.7) |  | | 8 (50.0) | 5 (62.5) |  | | | 63 (55.8) | 26 (45.6) |  | |
| T3/T4 | 20 (15.5) | 11 (16.9) |  | | 5 (31.3) | 1 (12.5) |  | | | 15 (13.3) | 10 (17.5) |  | |
| **cN stage** |  |  |  | |  |  |  | | |  |  |  | |
| N0 | 62 (48.1) | 36 (55.4) | 0.366 | | 7 (43.8) | 7 (87.5) | 0.116 | | | 55 (48.7) | 29 (50.9) | 0.498 | |
| N1 | 53 (41.1) | 20 (30.8) |  | | 7 (43.8) | 1 (12.5) |  | | | 46 (40.7) | 19 (33.3) |  | |
| N2/N3 | 14 (10.9) | 9 (13.8) |  | | 2 (12.5) | 0 (0.0) |  | | | 12 (10.6) | 9 (15.8) |  | |
| **Ki-67 index** (%) |  |  |  | |  |  |  | | |  |  |  | |
| ≤ 20 | 4 (3.1) | 1 (1.5) | 0.517 | | 1 (6.3) | 1 (12.5) | 0.602 | | | 3 (2.7) | 0 (0.0) | 0.215 | |
| > 20 | 125 (96.9) | 64 (98.5) |  | | 15 (93.8) | 7 (87.5) |  | | | 110 (97.3) | 57 (100.0) |  | |
| **Mean Ki-67 index** (%) | 63.1±20.3 | 65.0±19.4 | 0.530 | | 57.5±18.7 | 49.4±23.2 | 0.364 | | | 63.9±20.5 | 67.2±18.0 | 0.301 | |
| **Grade** |  |  |  | |  |  |  | | |  |  |  | |
| G2 | 15 (11.6) | 7 (10.8) | 0.859 | | 1 (6.3) | 2 (25.0) | 0.190 | | | 14 (12.4) | 5 (8.8) | 0.480 | |
| G3 | 114 (88.4) | 58 (89.2) |  | | 15 (93.8) | 6 (75.0) |  | | | 99 (87.6) | 52 (91.2) |  | |
| **pCR** |  |  |  | |  |  |  | | |  |  |  | |
| Yes | 63 (48.8) | 25 (38.5) | 0.171 | | 2 (12.5) | 0 (0.0) | 0.296 | | | 61 (54.0) | 25 (43.9) | 0.213 | |
| No | 66 (51.2) | 40 (61.5) |  | | 14 (87.5) | 8 (100.0) |  | | | 52 (46.0) | 32 (56.1) |  | |
| **Mean RCB score** | 1.207±1.425 | 1.429±1.497 | 0.327 | | 2.356±1.258 | 2.078±0.502 | 0.558 | | | 1.048±1.377 | 1.333±1.572 | 0.238 | |
| **Surgery type** |  |  |  | |  |  |  | | |  |  |  | |
| BCT | 78 (60.5) | 36 (55.4) | 0.497 | | 6 (37.5) | 6 (75.0) | 0.083 | | | 72 (63.7) | 30 (52.6) | 0.164 | |
| Mastectomy | 51 (39.5) | 29 (44.6) |  | | 10 (62.5) | 2 (25.0) |  | | | 41 (36.3) | 27 (47.4) |  | |
| **Adjuvant CT** (missing: 7) |  |  |  | |  |  |  | | |  |  |  | |
| Yes | 41 (32.5) | 19 (31.1) | 0.848 | | 10 (66.7) | 6 (75.0) | 0.679 | | | 31 (27.9) | 13 (24.5) | 0.646 | |
| No | 85 (67.5) | 42 (68.9) |  | | 5 (33.3) | 2 (25.0) |  | | | 80 (72.1) | 40 (75.5) |  | |
| **Adjuvant RT** (missing: 7) |  |  |  | |  |  |  | | |  |  |  | |
| Yes | 105 (83.3) | 48 (78.7) | 0.440 | | 12 (80.0) | 8 (100.0) | 0.175 | | | 93 (83.8) | 40 (75.5) | 0.204 | |
| No | 21 (16.7) | 13 (21.3) |  | | 3 (20.0) | 11 (47.8) |  | | | 18 (16.2) | 13 (24.5) |  | |

TNBC triple-negative breast cancer, ST special type, NST no special type, NAC neoadjuvant chemotherapy, pCR pathological complete response, RCB residual cancer burden, BCT breast conserving therapy, CT chemotherapy, RT radiotherapy.
