# Supplementary material for: Prognostic impact of HER2-low expression in triple-negative breast cancer of high-grade special histological type and no special type
Source: PLoS One. 2025 Jun 13;20(6):e0325715. doi: 10.1371/journal.pone.0325715 (PMC12165359; doi:10.1371/journal.pone.0325715)
Supplement: S8 Table — (DOCX) [file pone.0325715.s008.docx]

**S8 Table. Univariate and multivariate analyses of clinicopathological variables in non-NAC patients with high-grade TNBC ST and TNBC NST (n=294).**

| **Univariate** | **OS** | | | **DDFS** | | | **DFS** | | |
| --- | --- | --- | --- | --- | --- | --- | --- | --- | --- |
|  | **HR** | **95% CI** | ***p*-Value** | **HR** | **95% CI** | ***p*-Value** | **HR** | **95% CI** | ***p*-Value** |
| **Age** (years) |  |  |  |  |  |  |  |  |  |
| < 50 | 1 |  | **0.009** | 1 |  | 0.444 | 1 |  | 0.103 |
| ≥ 50 | 2.19 | 1.21-3.94 |  | 1.27 | 0.69-2.31 |  | 1.49 | 0.92-2.39 |  |
| **Year of diagnosis** |  |  |  |  |  |  |  |  |  |
| 2010-2017 | 1 |  | 0.239 | 1 |  | 0.406 | 1 |  | 0.142 |
| 2018-2023 | 1.42 | 0.79-2.54 |  | 1.29 | 0.71-2.36 |  | 1.44 | 0.89-2.34 |  |
| **TNBC subgroup** |  |  |  |  |  |  |  |  |  |
| NST | 1 |  | **0.018** | 1 |  | **<0.001** | 1 |  | **<0.001** |
| ST high-grade | 1.83 | 1.11-3.02 |  | 2.95 | 1.71-5.09 |  | 2.23 | 1.43-3.46 |  |
| **pT category** |  |  |  |  |  |  |  |  |  |
| T1 | 1 |  | **<0.001** | 1 |  | **<0.001** | 1 |  | **<0.001** |
| T2 | 2.63 | 1.51-4.57 |  | 2.53 | 1.32-4.86 |  | 2.10 | 1.31-3.36 |  |
| T3/T4 | 5.29 | 2.68-10.43 |  | 5.89 | 2.76-12.54 |  | 4.01 | 2.23-7.24 |  |
| **Nodal status** |  |  |  |  |  |  |  |  |  |
| N- | 1 |  | **<0.001** | 1 |  | **<0.001** | 1 |  | **<0.001** |
| N+ | 2.67 | 1.67-4.27 |  | 3.86 | 2.24-6.65 |  | 2.66 | 1.76-4.03 |  |
| **HER2 IHC score** |  |  |  |  |  |  |  |  |  |
| 0 | 1 |  | 0.400 | 1 |  | 0.695 | 1 |  | 0.755 |
| 1+/2+ | 0.80 | 0.48-1.34 |  | 0.89 | 0.50-1.59 |  | 0.93 | 0.60-1.45 |  |
| **Ki-67 index** (%) |  |  |  |  |  |  |  |  |  |
| ≤ 20 | 1 |  | 0.705 | 1 |  | 0.058 | 1 |  | 0.110 |
| > 20 | 0.84 | 0.33-2.12 |  | 0.44 | 0.19-1.03 |  | 0.55 | 0.27-1.14 |  |
| **Grade** |  |  |  |  |  |  |  |  |  |
| G2 | 1 |  | 0.656 | 1 |  | 0.357 | 1 |  | 0.492 |
| G3 | 1.27 | 0.45-3.55 |  | 0.65 | 0.26-1.63 |  | 0.76 | 0.35-1.66 |  |
| **Adjuvant CT** |  |  |  |  |  |  |  |  |  |
| Yes | 1 |  | **0.004** | 1 |  | **0.035** | 1 |  | **<0.001** |
| No | 2.11 | 1.26-3.52 |  | 1.90 | 1.05-3.46 |  | 2.18 | 1.39-3.43 |  |
| **Adjuvant RT** |  |  |  |  |  |  |  |  |  |
| Yes | 1 |  | **0.003** | 1 |  | 0.210 | 1 |  | **0.002** |
| No | 2.10 | 1.29-3.43 |  | 1.46 | 0.81-2.63 |  | 2.00 | 1.29-3.09 |  |
| **Multivariate** |  | **OS** |  |  | **DDFS** |  |  | **DFS** |  |
|  | **HR** | **95% CI** | ***p*-Value** | **HR** | **95% CI** | ***p*-Value** | **HR** | **95% CI** | ***p*-Value** |
| **Age** (years) |  |  |  |  |  |  |  |  |  |
| < 50 | 1 |  | 0.253 | - | - | - | 1 |  | 0.874 |
| ≥ 50 | 1.44 | 0.77-2.67 |  |  |  |  | 0.96 | 0.57-1.60 |  |
| **Year of diagnosis** |  |  |  |  |  |  |  |  |  |
| 2010-2017 | - | - | - | - | - | - | 1 |  | 0.135 |
| 2018-2023 |  |  |  |  |  |  | 1.47 | 0.89-2.42 |  |
| **TNBC subgroup** |  |  |  |  |  |  |  |  |  |
| NST | 1 |  | 0.281 | 1 |  | **0.002** | 1 |  | **0.003** |
| ST high-grade | 1.33 | 0.79-2.25 |  | 2.56 | 1.43-4.58 |  | 2.02 | 1.27-3.23 |  |
| **pT category** |  |  |  |  |  |  |  |  |  |
| T1 | 1 |  | **<0.001** | 1 |  | **0.004** | 1 |  | **<0.001** |
| T2 | 1.85 | 1.04-3.30 |  | 1.74 | 0.90-3.38 |  | 1.63 | 1.00-2.65 |  |
| T3/T4 | 5.02 | 2.45-10.29 |  | 3.87 | 1.75-8.58 |  | 3.52 | 1.87-6.64 |  |
| **Nodal status** |  |  |  |  |  |  |  |  |  |
| N- | 1 |  | **<0.001** | 1 |  | **<0.001** | 1 |  | **<0.001** |
| N+ | 2.90 | 1.79-4.71 |  | 4.11 | 2.34-7.23 |  | 2.99 | 1.94-4.60 |  |
| **Ki-67 index (%)** |  |  |  |  |  |  |  |  |  |
| ≤ 20 | - | - | - | 1 |  | 0.993 | 1 |  | 0.734 |
| > 20 |  |  |  | 1.00 | 0.40-2.50 |  | 1.15 | 0.52-2.51 |  |
| **Adjuvant CT** |  |  |  |  |  |  |  |  |  |
| Yes | 1 |  | 0.200 | 1 |  | **0.022** | 1 |  | **0.024** |
| No | 1.47 | 0.82-2.66 |  | 2.10 | 1.11-3.96 |  | 1.84 | 1.09-3.13 |  |
| **Adjuvant RT** |  |  |  |  |  |  |  |  |  |
| Yes | 1 |  | **0.004** | - | - | **-** | 1 |  | **0.003** |
| No | 2.31 | 1.31-4.06 |  |  |  |  | 2.14 | 1.30-3.53 |  |

TNBC triple-negative breast cancer, ST special type, NST no special type, NAC neoadjuvant chemotherapy, OS overall survival, DDFS distant disease-free survival, DFS disease-free survival, CT chemotherapy, RT radiotherapy.
