# Supplementary material for: Prognostic impact of HER2-low expression in triple-negative breast cancer of high-grade special histological type and no special type
Source: PLoS One. 2025 Jun 13;20(6):e0325715. doi: 10.1371/journal.pone.0325715 (PMC12165359; doi:10.1371/journal.pone.0325715)
Supplement: S9 Table — (DOCX) [file pone.0325715.s009.docx]

**S9 Table. Univariate and multivariate analyses of clinicopathological variables in non-NAC patients with high-grade TNBC ST (n=73).**

| **Univariate** | **OS** | | | **DDFS** | | | **DFS** | | |
| --- | --- | --- | --- | --- | --- | --- | --- | --- | --- |
|  | **HR** | **95% CI** | ***p*-Value** | **HR** | **95% CI** | ***p*-Value** | **HR** | **95% CI** | ***p*-Value** |
| **Age** (years) |  |  |  |  |  |  |  |  |  |
| < 50 | 1 |  | 0.325 | 1 |  | 0.737 | 1 |  | 0.313 |
| ≥ 50 | 1.73 | 0.58-5.18 |  | 1.19 | 0.43-3.25 |  | 1.60 | 0.64-3.96 |  |
| **Year of diagnosis** |  |  |  |  |  |  |  |  |  |
| 2010-2017 | 1 |  | 0.871 | 1 |  | 0.833 | 1 |  | 0.528 |
| 2018-2023 | 0.90 | 0.25-3.23 |  | 1.11 | 0.42-2.90 |  | 1.30 | 0.58-2.94 |  |
| **pT category** |  |  |  |  |  |  |  |  |  |
| T1 | 1 |  | **0.041** | 1 |  | **0.025** | 1 |  | **0.010** |
| T2 | 1.65 | 0.51-5.37 |  | 2.66 | 0.74-9.57 |  | 1.90 | 0.73-4.97 |  |
| T3/T4 | 4.15 | 1.25-13.79 |  | 5.92 | 1.56-22.37 |  | 4.46 | 1.63-12.22 |  |
| **Nodal status** |  |  |  |  |  |  |  |  |  |
| N- | 1 |  | **<0.001** | 1 |  | **<0.001** | 1 |  | **<0.001** |
| N+ | 4.44 | 1.84-10.74 |  | 5.01 | 2.08-12.09 |  | 4.25 | 2.01-8.97 |  |
| **HER2 IHC score** |  |  |  |  |  |  |  |  |  |
| 0 | 1 |  | 0.158 | 1 |  | 0.457 | 1 |  | 0.176 |
| 1+/2+ | 0.48 | 0.18-1.33 |  | 0.70 | 0.27-1.79 |  | 0.57 | 0.25-1.29 |  |
| **Ki-67 index** (%) |  |  |  |  |  |  |  |  |  |
| ≤ 20 | 1 |  | 0.820 | 1 |  | 0.448 | 1 |  | 0.579 |
| > 20 | 1.15 | 0.34-3.93 |  | 0.68 | 0.25-1.86 |  | 0.78 | 0.32-1.90 |  |
| **Grade** |  |  |  |  |  |  |  |  |  |
| G2 | 1 |  | 0.626 | 1 |  | 0.536 | 1 |  | 0.533 |
| G3 | 1.44 | 0.33-6.31 |  | 0.70 | 0.22-2.18 |  | 0.73 | 0.28-1.95 |  |
| **Adjuvant CT** |  |  |  |  |  |  |  |  |  |
| Yes | 1 |  | 0.764 | 1 |  | 0.419 | 1 |  | 0.106 |
| No | 0.85 | 0.28-2.52 |  | 1.48 | 0.57-3.81 |  | 1.92 | 0.87-4.23 |  |
| **Adjuvant RT** |  |  |  |  |  |  |  |  |  |
| Yes | 1 |  | 0.669 | 1 |  | 0.773 | 1 |  | 0.586 |
| No | 1.22 | 0.49-3.03 |  | 1.14 | 0.47-2.80 |  | 1.23 | 0.58-2.62 |  |
| **Multivariate** |  | **OS** |  |  | **DDFS** |  |  | **DFS** |  |
|  | **HR** | **95% CI** | ***p*-Value** | **HR** | **95% CI** | ***p*-Value** | **HR** | **95% CI** | ***p*-Value** |
| **pT category** |  |  |  |  |  |  |  |  |  |
| T1 | 1 |  | **0.029** | 1 |  | **0.031** | 1 |  | **0.037** |
| T2 | 1.19 | 0.35-3.97 |  | 2.31 | 0.64-8.36 |  | 1.95 | 0.73-5.22 |  |
| T3/T4 | 4.08 | 1.18-14.14 |  | 5.48 | 1.43-21.01 |  | 3.83 | 1.36-10.83 |  |
| **Nodal status** |  |  |  |  |  |  |  |  |  |
| N- | 1 |  | **<0.001** | 1 |  | **<0.001** | 1 |  | **<0.001** |
| N+ | 5.06 | 1.93-13.29 |  | 4.90 | 1.98-12.11 |  | 4.57 | 1.99-10.46 |  |
| **HER2 IHC score** |  |  |  |  |  |  |  |  |  |
| 0 | 1 |  | 0.945 | - | - | - | 1 |  | 0.877 |
| 1+/2+ | 0.96 | 0.32-2.91 |  |  |  |  | 0.93 | 0.37-2.33 |  |
| **Adjuvant CT** |  |  |  |  |  |  |  |  |  |
| Yes | - | - | - | - | - | - | 1 |  | **0.021** |
| No |  |  |  |  |  |  | 2.74 | 1.17-6.43 |  |

TNBC triple-negative breast cancer, ST special type, NAC neoadjuvant chemotherapy, OS overall survival, DDFS distant disease-free survival, DFS disease-free survival, CT chemotherapy, RT radiotherapy.
