# Supplementary material for: Prognostic impact of HER2-low expression in triple-negative breast cancer of high-grade special histological type and no special type
Source: PLoS One. 2025 Jun 13;20(6):e0325715. doi: 10.1371/journal.pone.0325715 (PMC12165359; doi:10.1371/journal.pone.0325715)
Supplement: S10 Table — (DOCX) [file pone.0325715.s010.docx]

**S10 Table. Univariate and multivariate analyses of clinicopathological variables in non-NAC patients with TNBC NST (n=221).**

| **Univariate** | **OS** | | | **DDFS** | | | **DFS** | | |
| --- | --- | --- | --- | --- | --- | --- | --- | --- | --- |
|  | **HR** | **95% CI** | ***p*-Value** | **HR** | **95% CI** | ***p*-Value** | **HR** | **95% CI** | ***p*-Value** |
| **Age** (years) |  |  |  |  |  |  |  |  |  |
| < 50 | 1 |  | **0.027** | 1 |  | 0.800 | 1 |  | 0.262 |
| ≥ 50 | 2.22 | 1.10-4.49 |  | 1.11 | 0.51-2.39 |  | 1.38 | 0.78-2.44 |  |
| **Year of diagnosis** |  |  |  |  |  |  |  |  |  |
| 2010-2017 | 1 |  | 0.124 | 1 |  | 0.349 | 1 |  | 0.157 |
| 2018-2023 | 1.69 | 0.87-3.30 |  | 1.45 | 0.67-3.16 |  | 1.55 | 0.84-2.85 |  |
| **pT category** |  |  |  |  |  |  |  |  |  |
| T1 | 1 |  | **<0.001** | 1 |  | **0.023** | 1 |  | **0.013** |
| T2 | 2.77 | 1.46-5.23 |  | 2.02 | 0.92-4.47 |  | 2.02 | 1.17-3.49 |  |
| T3/T4 | 4.60 | 1.87-11.30 |  | 4.18 | 1.45-12.07 |  | 2.73 | 1.17-6.38 |  |
| **Nodal status** |  |  |  |  |  |  |  |  |  |
| N- | 1 |  | **0.002** | 1 |  | **<0.001** | 1 |  | **<0.001** |
| N+ | 2.46 | 1.39-4.33 |  | 4.23 | 2.04-8.80 |  | 2.41 | 1.44-4.03 |  |
| **HER2 IHC score** |  |  |  |  |  |  |  |  |  |
| 0 | 1 |  | 0.716 | 1 |  | 0.851 | 1 |  | 0.631 |
| 1+/2+ | 0.89 | 0.48-1.65 |  | 0.93 | 0.43-2.00 |  | 1.14 | 0.66-1.96 |  |
| **Ki-67 index** (%) |  |  |  |  |  |  |  |  |  |
| ≤ 20 | 1 |  | 0.777 | 1 |  | 0.864 | 1 |  | 0.764 |
| > 20 | 1.33 | 0.18-9.66 |  | 0.84 | 0.11-6.17 |  | 0.81 | 0.20-3.30 |  |
| **Grade** |  |  |  |  |  |  |  |  |  |
| G2 | 1 |  | 0.394 | 1 |  | 0.623 | 1 |  | 0.607 |
| G3 | 2.37 | 0.33-17.21 |  | 1.65 | 0.22-12.12 |  | 1.45 | 0.35-5.95 |  |
| **Adjuvant CT** |  |  |  |  |  |  |  |  |  |
| Yes | 1 |  | **<0.001** | 1 |  | 0.051 | 1 |  | **0.003** |
| No | 2.88 | 1.59-5.20 |  | 2.18 | 1.00-4.76 |  | 2.31 | 1.33-4.02 |  |
| **Adjuvant RT** |  |  |  |  |  |  |  |  |  |
| Yes | 1 |  | **0.002** | 1 |  | 0.277 | 1 |  | **0.002** |
| No | 2.54 | 1.41-4.56 |  | 1.55 | 0.70-3.40 |  | 2.38 | 1.39-4.06 |  |
| **Multivariate** |  | **OS** |  |  | **DDFS** |  |  | **DFS** |  |
|  | **HR** | **95% CI** | ***p*-Value** | **HR** | **95% CI** | ***p*-Value** | **HR** | **95% CI** | ***p*-Value** |
| **Age** (years) |  |  |  |  |  |  |  |  |  |
| < 50 | 1 |  | 0.356 | - | - | - | - | - | - |
| ≥ 50 | 1.42 | 0.67-3.00 |  |  |  |  |  |  |  |
| **Year of diagnosis** |  |  |  |  |  |  | 1 |  | 0.187 |
| 2010-2017 | 1 |  | 0.286 | - | - | - | 1.51 | 0.82-2.80 |  |
| 2018-2023 | 1.45 | 0.73-2.89 |  |  |  |  |  |  |  |
| **pT category** |  |  |  |  |  |  |  |  |  |
| T1 | 1 |  | **0.003** | 1 |  | **0.049** | 1 |  | 0.051 |
| T2 | 1.86 | 0.95-3.61 |  | 1.38 | 0.62-3.10 |  | 1.50 | 0.84-2.66 |  |
| T3/T4 | 4.77 | 1.92-11.89 |  | 3.81 | 1.31-11.11 |  | 2.80 | 1.19-6.59 |  |
| **Nodal status** |  |  |  |  |  |  |  |  |  |
| N- | 1 |  | **<0.001** | 1 |  | **<0.001** | 1 |  | **<0.001** |
| N+ | 2.74 | 1.52-4.91 |  | 4.29 | 2.04-9.04 |  | 2.70 | 1.59-4.59 |  |
| **Adjuvant CT** |  |  |  |  |  |  |  |  |  |
| Yes | 1 |  | 0.055 | 1 |  | **0.018** | 1 |  | 0.100 |
| No | 2.04 | 0.98-4.22 |  | 2.69 | 1.19-6.10 |  | 1.72 | 0.90-3.29 |  |
| **Adjuvant RT** |  |  |  |  |  |  |  |  |  |
| Yes | 1 |  | **0.025** | - | - | **-** | 1 |  | **0.008** |
| No | 2.21 | 1.11-4.43 |  |  |  |  | 2.31 | 1.25-4.27 |  |

TNBC triple-negative breast cancer, NST no special type, NAC neoadjuvant chemotherapy, OS overall survival, DDFS distant disease-free survival, DFS disease-free survival, CT chemotherapy, RT radiotherapy.
