# Supplementary material for: Prognostic impact of HER2-low expression in triple-negative breast cancer of high-grade special histological type and no special type
Source: PLoS One. 2025 Jun 13;20(6):e0325715. doi: 10.1371/journal.pone.0325715 (PMC12165359; doi:10.1371/journal.pone.0325715)
Supplement: S11 Table — (DOCX) [file pone.0325715.s011.docx]

**S11 Table. Survival outcomes in patients with high-grade TNBC ST and TNBC NST.**

|  | 5-year OS  % (95% CI) | 5-year DDFS  % (95% CI) | 5-year DFS  % (95% CI) | No. of deaths by any cause during follow-up | No. of distant recurrences during follow-up |
| --- | --- | --- | --- | --- | --- |
| **Patients not receiving NAC** |  |  |  |  |  |
| *HER2 0 TNBC* |  |  |  |  |  |
| ST high-grade (n=50) | 80.0 (58.7-87.7) | 70.0 (41.4-75.2) | 62.0 (37.0-68.8) | 17 (34.0) | 16 (32.0) |
| NST (n=141) | 83.0 (73.2-87.4) | 86.5 (77.8-90.8) | 80.9 (71.4-86.0) | 33 (23.4) | 20 (14.2) |
| *HER2 1+/2+ TNBC* |  |  |  |  |  |
| ST high-grade (n=23) | 78.3 (47.4-92.0) | 73.9 (48.6-90.2) | 69.6 (45.0-86.6) | 6 (26.1) | 7 (30.4) |
| NST (n=80) | 83.8 (73.0-91.0) | 87.5 (76.2-93.8) | 76.3 (62.7-83.9) | 15 (18.8) | 10 (12.5) |
| **Patients receiving NAC** |  |  |  |  |  |
| *HER2 0 TNBC* |  |  |  |  |  |
| ST high-grade (n=14) | 78.6 (38.0-99.6) | 71.4 (41.4-94.0) | 57.1 (3.3-76.9) | 4 (28.6) | 4 (28.6) |
| NST (n=111) | 83.8 (69.1-87.5) | 82.0 (70.4-87.2) | 79.3 (66.5-84.5) | 22 (19.8) | 22 (19.8) |
| *HER2 1+/2+ TNBC* |  |  |  |  |  |
| ST high-grade (n=8) | 100.0 | 87.5 (53.5-113.1) | 87.5 (53.5-113.1) | 0 (0.0) | 1 (12.5) |
| NST (n=53) | 77.4 (58.9-85.9) | 77.4 (62.1-87.1) | 71.7 (53.3-81.1) | 14 (26.4) | 12 (22.6) |

TNBC triple-negative breast cancer, ST special type, NST no special type, NAC neoadjuvant chemotherapy.
