# Supplementary material for: Prognostic impact of HER2-low expression in triple-negative breast cancer of high-grade special histological type and no special type
Source: PLoS One. 2025 Jun 13;20(6):e0325715. doi: 10.1371/journal.pone.0325715 (PMC12165359; doi:10.1371/journal.pone.0325715)
Supplement: S12 Table — (DOCX) [file pone.0325715.s012.docx]

**S12 Table. Patient and tumor characteristics of pT-matched non-NAC HER2 0 high-grade TNBC ST and TNBC NST (n=100).**

|  | **HER2 0 TNBC** | | |  |
| --- | --- | --- | --- | --- |
| **Variable** | **Overall (n=100)** | **NST (n=50)** | **ST high-grade (n=50)** |  |
|  | **N (%)** | **N (%)** | **N (%)** | *p*-Value |
| **Age group** (years) |  |  |  |  |
| < 50 | 23 (23.0) | 13 (26.0) | 10 (20.0) | 0.476 |
| ≥ 50 | 77 (77.0) | 37 (74.0) | 40 (80.0) |  |
| **Mean age** (years) | 59.6±14.7 | 57.7±14.1 | 61.4±15.2 | 0.203 |
| **Year of diagnosis** |  |  |  |  |
| 2010-2017 | 63 (63.0) | 35 (70.0) | 28 (56.0) | 0.147 |
| 2018-2023 | 37 (37.0) | 15 (30.0) | 22 (44.0) |  |
| **pT category** |  |  |  |  |
| T1 | 36 (36.0) | 18 (36.0) | 18 (36.0) | 1.000 |
| T2 | 38 (38.0) | 19 (38.0) | 19 (38.0) |  |
| T3/T4 | 26 (26.0) | 13 (26.0) | 13 (26.0) |  |
| **Nodal status** |  |  |  |  |
| N- | 70 (70.0) | 37 (74.0) | 33 (66.0) | 0.383 |
| N+ | 30 (30.0) | 13 (26.0) | 17 (34.0) |  |
| **Grade** |  |  |  |  |
| G2 | 7 (7.0) | 2 (4.0) | 5 (11.9) | 0.154 |
| G3 | 85 (85.0) | 48 (96.0) | 37 (88.1) |  |
| **Ki-67 index** (%) |  |  |  |  |
| ≤ 20 | 9 (9.0) | 2 (4.0) | 7 (14.0) | 0.081 |
| > 20 | 91 (91.0) | 48 (96.0) | 43 (86.0) |  |
| **Mean Ki-67 index** (%) | 54.0±22.7 | 57.4±20.0 | 50.7±24.8 | 0.137 |
| **Grade** |  |  |  |  |
| G2 | 15 (11.6) | 14 (12.4) | 1 (6.3) | 0.473 |
| G3 | 114 (88.4) | 99 (87.6) | 15 (93.8) |  |
| **Surgery type** |  |  |  |  |
| BCT | 60 (60.0) | 34 (68.0) | 26 (52.0) | 0.102 |
| Mastectomy | 40 (40.0) | 16 (32.0) | 24 (48.0) |  |
| **Adjuvant CT** |  |  |  |  |
| Yes | 75 (75.0) | 37 (74.0) | 38 (76.0) | 0.817 |
| No | 25 (25.0) | 13 (26.0) | 12 (25.0) |  |
| **Adjuvant RT** |  |  |  |  |
| Yes | 79 (79.0) | 38 (76.0) | 41 (82.0) | 0.461 |
| No | 21 (21.0) | 12 (24.0) | 9 (18.0) |  |

NAC neoadjuvant chemotherapy, TNBC triple-negative breast cancer, ST special type, NST no special type, BCT breast conserving therapy, CT chemotherapy, RT radiotherapy.
