# Supplementary material for: Prognostic impact of HER2-low expression in triple-negative breast cancer of high-grade special histological type and no special type
Source: PLoS One. 2025 Jun 13;20(6):e0325715. doi: 10.1371/journal.pone.0325715 (PMC12165359; doi:10.1371/journal.pone.0325715)
Supplement: S13 Table — (DOCX) [file pone.0325715.s013.docx]

**S13 Table. Univariate and multivariate analyses of clinicopathological variables in NAC-treated patients with high-grade TNBC ST and TNBC NST (n=186).**

| **Univariate** | **OS** | | | **DDFS** | | | | | | **DFS** | | | | | |
| --- | --- | --- | --- | --- | --- | --- | --- | --- | --- | --- | --- | --- | --- | --- | --- |
|  | **HR** | **95% CI** | ***p*-Value** | | **HR** | **95% CI** | | ***p*-Value** | **HR** | | | | **95% CI** | | ***p*-Value** |
| **Age** (years) |  |  |  | |  |  | |  |  | | | |  | |  |
| < 50 | 1 |  | 0.705 | | 1 |  | | 0.170 | 1 | | | |  | | 0.502 |
| ≥ 50 | 0.88 | 0.45-1.71 |  | | 0.60 | 0.29-1.24 | |  | 0.82 | | | | 0.46-1.46 | |  |
| **Year of diagnosis** |  |  |  | |  |  | |  |  | | | |  | |  |
| 2010-2017 | 1 |  | 0.267 | | 1 |  | | 0.148 | 1 | | | |  | | 0.136 |
| 2018-2023 | 0.65 | 0.31-1.39 |  | | 0.61 | 0.31-1.20 | |  | 0.63 | | | | 0.34-1.16 | |  |
| **TNBC subgroup** |  |  |  | |  |  | |  |  | | | |  | |  |
| NST | 1 |  | 0.974 | | 1 |  | | 0.574 | 1 | | | |  | | 0.284 |
| ST high-grade | 0.98 | 0.35-2.76 |  | | 1.31 | 0.51-3.36 | |  | 1.55 | | | | 0.70-3.45 | |  |
| **ypT category** |  |  |  | |  |  | |  |  | | | |  | |  |
| T0 | 1 |  | **<0.001** | | 1 |  | | **<0.001** | 1 | | | |  | | **<0.001** |
| T1/T2 | 3.47 | 1.48-8.13 |  | | 4.20 | 1.69-10.41 | |  | 3.73 | | | | 1.83-7.62 | |  |
| T3/T4 | 24.29 | 9.30-63.47 |  | | 28.87 | 10.53-79.15 | |  | 16.57 | | | | 6.95-39.51 | |  |
| **cT stage** |  |  |  | |  |  | |  |  | | | |  | |  |
| T1/T2 | 1 |  | **<0.001** | | 1 |  | | **<0.001** | 1 | | | |  | | **<0.001** |
| T3/T4 | 5.09 | 2.69-9.62 |  | | 5.41 | 2.82-10.40 | |  | 3.99 | | | | 2.25-7.10 | |  |
| **Nodal status** (post-NAC) |  |  |  | |  |  | |  |  | | | |  | |  |
| N- | 1 |  | **<0.001** | | 1 |  | | **<0.001** | 1 | | | |  | | **<0.001** |
| N+ | 5.62 | 3.00-10.50 |  | | 6.27 | 3.29-11.98 | |  | 3.77 | | | | 2.17-6.53 | |  |
| **Nodal status** (pre-NAC) |  |  |  | |  |  | |  |  | | | |  | |  |
| N- | 1 |  | **<0.001** | | 1 |  | | **<0.001** | 1 | | | |  | | **0.008** |
| N+ | 4.26 | 1.96-9.27 |  | | 4.32 | 1.98-9.44 | |  | 2.19 | | | | 1.23-3.92 | |  |
| **HER2 IHC score** |  |  |  | |  |  | |  |  | | | |  | |  |
| 0 | 1 |  | 0.332 | | 1 |  | | 0.607 | 1 | | | |  | | 0.291 |
| 1+/2+ | 1.38 | 0.72-2.65 |  | | 1.19 | 0.61-2.33 | |  | 1.36 | | | | 0.77-2.39 | |  |
| **Ki-67 index** (%) |  |  |  | |  |  | |  |  | | | |  | |  |
| ≤ 20 | 1 |  | 0.188 | | 1 |  | | 0.250 | 1 | | | |  | | 0.405 |
| > 20 | 0.38 | 0.09-1.60 |  | | 0.43 | 0.10-1.80 | |  | 0.55 | | | | 0.13-2.26 | |  |
| **Grade** |  |  |  | |  |  | |  |  | | | |  | |  |
| G2 | 1 |  | 0.609 | | 1 |  | | 0.997 | 1 | | | |  | | 0.580 |
| G3 | 1.31 | 0.47-3.68 |  | | 1.00 | 0.39-2.57 | |  | 0.81 | | | | 0.38-1.72 | |  |
| **pCR** |  |  |  | |  |  | |  |  | | | |  | |  |
| Yes | 1 |  | **<0.001** | | 1 |  | | **<0.001** | 1 | | | |  | | **<0.001** |
| No | 4.93 | 2.07-11.75 |  | | 6.10 | 2.38-15.64 | |  | 4.50 | | | | 2.19-9.23 | |  |
| **Adjuvant CT** |  |  |  | |  |  | |  |  | | | |  | |  |
| Yes | 1 |  | 0.112 | | 1 |  | | 0.076 | 1 | | | |  | | **0.002** |
| No | 0.59 | 0.31-1.13 |  | | 0.56 | 0.29-1.06 | |  | 0.41 | | | | 0.24-0.72 | |  |
| **Adjuvant RT** |  |  |  | |  |  | |  |  | | | |  | |  |
| Yes | 1 |  | 0.466 | | 1 |  | | 0.168 | 1 | | | |  | | 0.773 |
| No | 0.72 | 0.30-1.73 |  | | 0.48 | 0.17-1.36 | |  | 0.90 | | | | 0.44-1.85 | |  |
| **Multivariate** |  | **OS** |  | |  | **DDFS** |  | | | |  | **DFS** | |  | |
|  | **HR** | **95% CI** | ***p*-Value** | | **HR** | **95% CI** | | ***p*-Value** | **HR** | | | | **95% CI** | | ***p*-Value** |
| **Age** (years) |  |  |  | |  |  | |  |  | | | |  | |  |
| < 50 | - | - | - | | 1 |  | | 0.222 | - | | | | - | | - |
| ≥ 50 |  |  |  | | 0.62 | 0.29-1.33 | |  |  | | | |  | |  |
| **Year of diagnosis** |  |  |  | |  |  | |  |  | | | |  | |  |
| 2010-2017 | - | - | - | | 1 |  | | 0.938 | 1 | | | |  | | 0.253 |
| 2018-2023 |  |  |  | | 0.97 | 0.44-2.15 | |  | 0.68 | | | | 0.35-1.32 | |  |
| **ypT category** |  |  |  | |  |  | |  |  | | | |  | |  |
| T0 | 1 |  | 0.056 | | 1 |  | | 0.114 | 1 | | | |  | | 0.163 |
| T1/T2 | 2.65 | 0.58-12.17 |  | | 3.22 | 0.57-18.18 | |  | 3.11 | | | | 0.65-14.85 | |  |
| T3/T4 | 8.52 | 1.36-53.38 |  | | 8.51 | 1.08-67.18 | |  | 5.61 | | | | 0.94-33.29 | |  |
| **cT stage** |  |  |  | |  |  | |  |  | | | |  | |  |
| T1/T2 | 1 |  | 0.467 | | 1 |  | | 0.646 | 1 | | | |  | | 0.261 |
| T3/T4 | 1.46 | 0.53-4.04 |  | | 1.29 | 0.43-3.89 | |  | 1.63 | | | | 0.70-3.79 | |  |
| **Nodal status** (post-NAC) |  |  |  | |  |  | |  |  | | | |  | |  |
| N- | 1 |  | 0.168 | | 1 |  | | 0.102 | 1 | | | |  | | 0.147 |
| N+ | 1.91 | 0.76-4.77 |  | | 2.35 | 0.84-6.52 | |  | 1.86 | | | | 0.81-4.28 | |  |
| **Nodal status** (pre-NAC) |  |  |  | |  |  | |  |  | | | |  | |  |
| N- | 1 |  | 0.222 | | 1 |  | | 0.335 | 1 | | | |  | | 0.935 |
| N+ | 1.83 | 0.69-4.82 |  | | 1.65 | 0.60-4.57 | |  | 1.03 | | | | 0.48-2.23 | |  |
| **Ki-67 index** (%) |  |  |  | |  |  | |  |  | | | |  | |  |
| ≤ 20 | 1 |  | 0.199 | | - | - | | - | - | | | | - | | - |
| > 20 | 0.37 | 0.08-1.68 |  | |  |  | |  |  | | | |  | |  |
| **pCR** |  |  |  | |  |  | |  |  | | | |  | |  |
| Yes | 1 |  | 0.838 | | 1 |  | | 0.889 | 1 | | | |  | | 0.919 |
| No | 1.19 | 0.23-6.30 |  | | 1.15 | 0.17-7.84 | |  | 0.92 | | | | 0.17-4.93 | |  |
| **Adjuvant CT** |  |  |  | |  |  | |  |  | | | |  | |  |
| Yes | 1 |  | 0.296 | | 1 |  | | 0.432 | 1 | | | |  | | 0.546 |
| No | 1.46 | 0.72-2.99 |  | | 1.36 | 0.63-2.94 | |  | 0.82 | | | | 0.42-1.58 | |  |
| **Adjuvant RT** |  |  |  | |  |  | |  |  | | | |  | |  |
| Yes | - | - | - | | 1 |  | | 0.745 | - | | | | - | | - |
| No |  |  |  | | 0.83 | 0.27-2.53 | |  |  | | | |  | |  |

TNBC triple-negative breast cancer, NST no special type, ST special type, NAC neoadjuvant chemotherapy, OS overall survival, DDFS distant disease-free survival, DFS disease-free survival, pCR pathological complete response, CT chemotherapy, RT radiotherapy.
