# Supplementary material for: Prognostic impact of HER2-low expression in triple-negative breast cancer of high-grade special histological type and no special type
Source: PLoS One. 2025 Jun 13;20(6):e0325715. doi: 10.1371/journal.pone.0325715 (PMC12165359; doi:10.1371/journal.pone.0325715)
Supplement: S14 Table — (DOCX) [file pone.0325715.s014.docx]

**S14 Table. Univariate and multivariate analyses of clinicopathological variables in NAC-treated patients with TNBC NST (n=164).**

| **Univariate** | **OS** | | | | **DDFS** | | | | | | | | **DFS** | | | | | |
| --- | --- | --- | --- | --- | --- | --- | --- | --- | --- | --- | --- | --- | --- | --- | --- | --- | --- | --- |
|  | **HR** | **95% CI** | | ***p*-Value** | | | **HR** | | **95% CI** | | ***p*-Value** | **HR** | | | | **95% CI** | | ***p*-Value** |
| **Age** (years) |  |  | |  | | |  | |  | |  |  | | | |  | |  |
| < 50 | 1 |  | | 0.818 | | | 1 | |  | | 0.277 | 1 | | | |  | | 0.953 |
| ≥ 50 | 0.92 | 0.46-1.84 | |  | | | 0.65 | | 0.30-1.41 | |  | 0.98 | | | | 0.53-1.81 | |  |
| **Year of diagnosis** |  |  | |  | | |  | |  | |  |  | | | |  | |  |
| 2010-2017 | 1 |  | | 0.382 | | | 1 | |  | | 0.260 | 1 | | | |  | | 0.333 |
| 2018-2023 | 0.71 | 0.33-1.53 | |  | | | 0.66 | | 0.32-1.36 | |  | 0.72 | | | | 0.38-1.39 | |  |
| **ypT category** |  |  | |  | | |  | |  | |  |  | | | |  | |  |
| T0 | 1 |  | | **<0.001** | | | 1 | |  | | **<0.001** | 1 | | | |  | | **<0.001** |
| T1/T2 | 3.32 | 1.38-7.95 | |  | | | 3.70 | | 1.45-9.47 | |  | 3.25 | | | | 1.55-6.81 | |  |
| T3/T4 | 29.56 | 11.20-78.02 | |  | | | 35.46 | | 12.83-97.99 | |  | 20.36 | | | | 8.49-48.86 | |  |
| **cT stage** |  |  | |  | | |  | |  | |  |  | | | |  | |  |
| T1/T2 | 1 |  | | **<0.001** | | | 1 | |  | | **<0.001** | 1 | | | |  | | **<0.001** |
| T3/T4 | 5.73 | 2.93-11.17 | |  | | | 6.46 | | 3.23-12.93 | |  | 4.86 | | | | 2.65-8.93 | |  |
| **Nodal status** (post-NAC) |  |  | |  | | |  | |  | |  |  | | | |  | |  |
| N- | 1 |  | | **<0.001** | | | 1 | |  | | **<0.001** | 1 | | | |  | | **<0.001** |
| N+ | 6.80 | 3.49-13.23 | |  | | | 8.38 | | 4.11-17.11 | |  | 4.76 | | | | 2.65-8.58 | |  |
| **Nodal status** (pre-NAC) |  |  | |  | | |  | |  | |  |  | | | |  | |  |
| N- | 1 |  | | **<0.001** | | | 1 | |  | | **<0.001** | 1 | | | |  | | **0.002** |
| N+ | 5.17 | 2.15-12.44 | |  | | | 6.27 | | 2.42-16.26 | |  | 2.86 | | | | 1.47-5.54 | |  |
| **HER2 IHC score** |  |  | |  | | |  | |  | |  |  | | | |  | |  |
| 0 | 1 |  | | 0.214 | | | 1 | |  | | 0.499 | 1 | | | |  | | 0.151 |
| 1+/2+ | 1.53 | 0.78-2.99 | |  | | | 1.28 | | 0.63-2.60 | |  | 1.55 | | | | 0.85-2.81 | |  |
| **Ki-67 index** (%) |  |  | |  | | |  | |  | |  |  | | | |  | |  |
| ≤ 20 | 1 |  | | 0.670 | | | 1 | |  | | 0.646 | 1 | | | |  | | 0.792 |
| > 20 | 0.65 | 0.09-4.75 | |  | | | 0.63 | | 0.09-4.59 | |  | 0.77 | | | | 0.11-5.57 | |  |
| **Grade** |  |  | |  | | |  | |  | |  |  | | | |  | |  |
| G2 | 1 |  | | 0.374 | | | 1 | |  | | 0.446 | 1 | | | |  | | 0.981 |
| G3 | 1.71 | 0.52-5.58 | |  | | | 1.59 | | 0.48-5.20 | |  | 0.99 | | | | 0.42-2.34 | |  |
| **pCR** |  |  | |  | | |  | |  | |  |  | | | |  | |  |
| Yes | 1 |  | | **<0.001** | | | 1 | |  | | **<0.001** | 1 | | | |  | | **<0.001** |
| No | 5.11 | 2.12-12.28 | |  | | | 6.06 | | 2.34-15.70 | |  | 4.29 | | | | 2.07-8.92 | |  |
| **Adjuvant CT** |  |  | |  | | |  | |  | |  |  | | | |  | |  |
| Yes | 1 |  | | 0.167 | | | 1 | |  | | 0.061 | 1 | | | |  | | **0.002** |
| No | 0.61 | 0.30-1.23 | |  | | | 0.51 | | 0.26-1.03 | |  | 0.38 | | | | 0.21-0.69 | |  |
| **Adjuvant RT** |  |  | |  | | |  | |  | |  |  | | | |  | |  |
| Yes | 1 |  | | 0.368 | | | 1 | |  | | 0.132 | 1 | | | |  | | 0.466 |
| No | 0.65 | 0.25-1.67 | |  | | | 0.40 | | 0.12-1.32 | |  | 0.74 | | | | 0.33-1.66 | |  |
| **Multivariate** |  | **OS** |  | | |  | | **DDFS** | |  | | | |  | **DFS** | |  | |
|  | **HR** | **95% CI** | | ***p*-Value** | | | **HR** | | **95% CI** | | ***p*-Value** | **HR** | | | | **95% CI** | | ***p*-Value** |
| **ypT category** |  |  | |  | | |  | |  | |  |  | | | |  | |  |
| T0 | 1 |  | | **0.035** | | | 1 | |  | | 0.047 | 1 | | | |  | | 0.147 |
| T1/T2 | 2.17 | 0.50-9.54 | |  | | | 2.70 | | 0.56-13.09 | |  | 2.38 | | | | 0.51-10.99 | |  |
| T3/T4 | 9.48 | 1.50-59.93 | |  | | | 11.28 | | 1.52-83.74 | |  | 5.52 | | | | 0.93-32.78 | |  |
| **cT stage** |  |  | |  | | |  | |  | |  |  | | | |  | |  |
| T1/T2 | 1 |  | | 0.765 | | | 1 | |  | | 0.935 | 1 | | | |  | | 0.268 |
| T3/T4 | 1.19 | 0.39-3.64 | |  | | | 1.05 | | 0.30-3.71 | |  | 1.70 | | | | 0.67-4.34 | |  |
| **Nodal status** (post-NAC) |  |  | |  | | |  | |  | |  |  | | | |  | |  |
| N- | 1 |  | | 0.107 | | | 1 | |  | | 0.141 | 1 | | | |  | | 0.230 |
| N+ | 2.21 | 0.84-5.77 | |  | | | 2.28 | | 0.76-6.83 | |  | 1.75 | | | | 0.70-4.36 | |  |
| **Nodal status** (pre-NAC) |  |  | |  | | |  | |  | |  |  | | | |  | |  |
| N- | 1 |  | | 0.200 | | | 1 | |  | | 0.151 | 1 | | | |  | | 0.536 |
| N+ | 2.02 | 0.69-5.94 | |  | | | 2.37 | | 0.73-7.66 | |  | 1.32 | | | | 0.55-3.13 | |  |
| **HER2 IHC score** |  |  | |  | | |  | |  | |  |  | | | |  | |  |
| 0 | - | - | | - | | | - | | - | | - | 1 | | | |  | | 0.360 |
| 1+/2+ |  |  | |  | | |  | |  | |  | 1.35 | | | | 0.71-2.57 | |  |
| **pCR** |  |  | |  | | |  | |  | |  |  | | | |  | |  |
| Yes | 1 |  | | 0.744 | | | 1 | |  | | 0.923 | 1 | | | |  | | 0.984 |
| No | 1.31 | 0.26-6.64 | |  | | | 1.09 | | 0.19-6.44 | |  | 0.98 | | | | 0.19-5.13 | |  |
| **Adjuvant CT** |  |  | |  | | |  | |  | |  |  | | | |  | |  |
| Yes | 1 |  | | 0.251 | | | 1 | |  | | 0.645 | 1 | | | |  | | 0.435 |
| No | 1.56 | 0.73-3.31 | |  | | | 1.19 | | 0.56-2.53 | |  | 0.76 | | | | 0.38-1.52 | |  |
| **Adjuvant RT** |  |  | |  | | |  | |  | |  |  | | | |  | |  |
| Yes | - | - | | - | | | 1 | |  | | 0.756 | - | | | | - | | - |
| No |  |  | |  | | | 0.82 | | 0.23-2.94 | |  |  | | | |  | |  |

TNBC triple-negative breast cancer, NST no special type, NAC neoadjuvant chemotherapy, OS overall survival, DDFS distant disease-free survival, DFS disease-free survival, pCR pathological complete response, CT chemotherapy, RT radiotherapy.
