# Supplementary material for: Prognostic impact of HER2-low expression in triple-negative breast cancer of high-grade special histological type and no special type
Source: PLoS One. 2025 Jun 13;20(6):e0325715. doi: 10.1371/journal.pone.0325715 (PMC12165359; doi:10.1371/journal.pone.0325715)
Supplement: S15 Table — (DOCX) [file pone.0325715.s015.docx]

**S15 Table. Univariate and multivariate analyses of clinicopathological variables in NAC-treated patients with HER2 0 high-grade TNBC ST and TNBC NST (n=125).**

| **Univariate** | **OS** | | | | **DDFS** | | | | | | | | | | **DFS** | | | | | | | |
| --- | --- | --- | --- | --- | --- | --- | --- | --- | --- | --- | --- | --- | --- | --- | --- | --- | --- | --- | --- | --- | --- | --- |
|  | **HR** | **95% CI** | | ***p*-Value** | | | **HR** | | **95% CI** | | | ***p*-Value** | | **HR** | | | | | **95% CI** | | | ***p*-Value** |
| **Age** (years) |  |  | |  | | |  | |  | | |  | |  | | | | |  | | |  |
| < 50 | 1 |  | | 0.956 | | | 1 | |  | | | 0.599 | | 1 | | | | |  | | | 0.452 |
| ≥ 50 | 0.98 | 0.43-2.25 | |  | | | 0.79 | | 0.33-1.89 | | |  | | 0.75 | | | | | 0.35-1.60 | | |  |
| **Year of diagnosis** |  |  | |  | | |  | |  | | |  | |  | | | | |  | | |  |
| 2010-2017 | 1 |  | | 0.272 | | | 1 | |  | | | 0.163 | | 1 | | | | |  | | | 0.097 |
| 2018-2023 | 0.57 | 0.21-1.56 | |  | | | 0.53 | | 0.22-1.29 | | |  | | 0.50 | | | | | 0.22-1.13 | | |  |
| **TNBC subgroup** |  |  | |  | | |  | |  | | |  | |  | | | | |  | | |  |
| NST | 1 |  | | 0.592 | | | 1 | |  | | | 0.413 | | 1 | | | | |  | | | 0.101 |
| ST high-grade | 1.34 | 0.46-3.89 | |  | | | 1.56 | | 0.54-4.56 | | |  | | 2.10 | | | | | 0.87-5.11 | | |  |
| **ypT category** |  |  | |  | | |  | |  | | |  | |  | | | | |  | | |  |
| T0 | 1 |  | | **0.005** | | | 1 | |  | | | **0.001** | | 1 | | | | |  | | | **0.002** |
| T1/T2 | 3.36 | 1.32-8.55 | |  | | | 4.54 | | 1.68-12.32 | | |  | | 3.67 | | | | | 1.63-8.25 | | |  |
| T3/T4 | 8.69 | 2.16-34.91 | |  | | | 11.80 | | 2.81-49.55 | | |  | | 6.37 | | | | | 1.68-24.12 | | |  |
| **cT stage** |  |  | |  | | |  | |  | | |  | |  | | | | |  | | |  |
| T1/T2 | 1 |  | | **0.015** | | | 1 | |  | | | **0.009** | | 1 | | | | |  | | | **0.009** |
| T3/T4 | 2.94 | 1.23-7.01 | |  | | | 3.23 | | 1.34-7.75 | | |  | | 2.78 | | | | | 1.29-5.99 | | |  |
| **Nodal status** (post-NAC) |  |  | |  | | |  | |  | | |  | |  | | | | |  | | |  |
| N- | 1 |  | | **<0.001** | | | 1 | |  | | | **<0.001** | | 1 | | | | |  | | | **0.002** |
| N+ | 4.04 | 1.85-8.86 | |  | | | 4.83 | | 2.18-10.67 | | |  | | 3.12 | | | | | 1.52-6.41 | | |  |
| **Nodal status** (pre-NAC) |  |  | |  | | |  | |  | | |  | |  | | | | |  | | |  |
| N- | 1 |  | | **0.007** | | | 1 | |  | | | **0.007** | | 1 | | | | |  | | | 0.056 |
| N+ | 3.52 | 1.41-8.79 | |  | | | 3.51 | | 1.40-8.80 | | |  | | 2.03 | | | | | 0.98-4.18 | | |  |
| **Ki-67 index** (%) |  |  | |  | | |  | |  | | |  | |  | | | | |  | | |  |
| ≤ 20 | 1 |  | | 0.143 | | | 1 | |  | | | 0.200 | | 1 | | | | |  | | | 0.312 |
| > 20 | 0.34 | 0.08-1.44 | |  | | | 0.39 | | 0.09-1.65 | | |  | | 0.48 | | | | | 0.11-2.01 | | |  |
| **Grade** |  |  | |  | | |  | |  | | |  | |  | | | | |  | | |  |
| G2 | 1 |  | | 0.770 | | | 1 | |  | | | 0.772 | | 1 | | | | |  | | | 0.814 |
| G3 | 1.20 | 0.36-3.99 | |  | | | 1.20 | | 0.36-4.00 | | |  | | 0.89 | | | | | 0.34-2.31 | | |  |
| **pCR** |  |  | |  | | |  | |  | | |  | |  | | | | |  | | |  |
| Yes | 1 |  | | **0.005** | | | 1 | |  | | | **0.001** | | 1 | | | | |  | | | **0.001** |
| No | 4.10 | 1.54-10.89 | |  | | | 5.68 | | 1.95-16.57 | | |  | | 4.04 | | | | | 1.75-9.32 | | |  |
| **Adjuvant CT** |  |  | |  | | |  | |  | | |  | |  | | | | |  | | |  |
| Yes | 1 |  | | 0.061 | | | 1 | |  | | | **0.031** | | 1 | | | | |  | | | **0.002** |
| No | 0.47 | 0.21-1.04 | |  | | | 0.42 | | 0.19-0.93 | | |  | | 0.34 | | | | | 0.17-0.67 | | |  |
| **Adjuvant RT** |  |  | |  | | |  | |  | | |  | |  | | | | |  | | |  |
| Yes | 1 |  | | 0.131 | | | 1 | |  | | | 0.155 | | 1 | | | | |  | | | 0.157 |
| No | 0.33 | 0.08-1.39 | |  | | | 0.35 | | 0.08-1.49 | | |  | | 0.42 | | | | | 0.13-1.39 | | |  |
| **Multivariate** |  | **OS** |  | | |  | | **DDFS** | |  | | | | | |  | **DFS** | | |  | | |
|  | **HR** | **95% CI** | ***p*-Value** | | | **HR** | | **95% CI** | | | ***p*-Value** | | **HR** | | | | | **95% CI** | | | ***p*-Value** | |
| **Year of diagnosis** |  |  | |  | | |  | |  | | |  | |  | | | | |  | | |  |
| 2010-2017 | - | - | | - | | | 1 | |  | | | 0.112 | | 1 | | | | |  | | | **0.041** |
| 2018-2023 |  |  | |  | | | 0.43 | | 0.15-1.22 | | |  | | 0.37 | | | | | 0.15-0.96 | | |  |
| **TNBC subgroup** |  |  | |  | | |  | |  | | |  | |  | | | | |  | | |  |
| NST | - | - | | - | | | - | | - | | | - | | 1 | | | | |  | | | 0.756 |
| ST high-grade |  |  | |  | | |  | |  | | |  | | 1.16 | | | | | 0.45-3.05 | | |  |
| **ypT category** |  |  | |  | | |  | |  | | |  | |  | | | | |  | | |  |
| T0 | 1 |  | | 0.696 | | | 1 | |  | | | 0.516 | | 1 | | | | |  | | | 0.384 |
| T1/T2 | 2.50 | 0.30-20.60 | |  | | | 3.24 | | 0.40-26.26 | | |  | | 3.07 | | | | | 0.37-25.48 | | |  |
| T3/T4 | 2.40 | 0.18-32.68 | |  | | | 2.18 | | 0.17-28.76 | | |  | | 1.26 | | | | | 0.11-15.07 | | |  |
| **cT stage** |  |  | |  | | |  | |  | | |  | |  | | | | |  | | |  |
| T1/T2 | 1 |  | | 0.375 | | | 1 | |  | | | 0.259 | | 1 | | | | |  | | | 0.163 |
| T3/T4 | 1.70 | 0.53-5.52 | |  | | | 1.97 | | 0.61-6.36 | | |  | | 1.99 | | | | | 0.76-5.23 | | |  |
| **Nodal status** (post-NAC) |  |  | |  | | |  | |  | | |  | |  | | | | |  | | |  |
| N- | 1 |  | | 0.491 | | | 1 | |  | | | 0.089 | | 1 | | | | |  | | | 0.142 |
| N+ | 1.54 | 0.45-5.31 | |  | | | 2.96 | | 0.85-10.31 | | |  | | 2.33 | | | | | 0.76-7.17 | | |  |
| **Nodal status** (pre-NAC) |  |  | |  | | |  | |  | | |  | |  | | | | |  | | |  |
| N- | 1 |  | | 0.263 | | | 1 | |  | | | 0.454 | | 1 | | | | |  | | | 0.753 |
| N+ | 1.86 | 0.63-5.52 | |  | | | 1.54 | | 0.50-4.79 | | |  | | 1.16 | | | | | 0.47-2.86 | | |  |
| **Ki-67 index** (%) |  |  | |  | | |  | |  | | |  | |  | | | | |  | | |  |
| ≤ 20 | 1 |  | | 0.156 | | | - | | - | | | - | | - | | | | | - | | | **-** |
| > 20 | 0.30 | 0.06-1.59 | |  | | |  | |  | | |  | |  | | | | |  | | |  |
| **pCR** |  |  | |  | | |  | |  | | |  | |  | | | | |  | | |  |
| Yes | 1 |  | | 0.904 | | | 1 | |  | | | 0.955 | | 1 | | | | |  | | | 0.735 |
| No | 1.16 | 0.11-12.36 | |  | | | 0.93 | | 0.08-10.84 | | |  | | 0.66 | | | | | 0.06-7.10 | | |  |
| **Adjuvant CT** |  |  | |  | | |  | |  | | |  | |  | | | | |  | | |  |
| Yes | 1 |  | | 0.863 | | | 1 | |  | | | 0.961 | | 1 | | | | |  | | | 0.242 |
| No | 1.10 | 0.39-3.06 | |  | | | 1.03 | | 0.36-2.94 | | |  | | 0.58 | | | | | 0.23-1.45 | | |  |
| **Adjuvant RT** |  |  | |  | | |  | |  | | |  | |  | | | | |  | | |  |
| Yes | 1 |  | | 0.182 | | | 1 | | - | | | 0.392 | | 1 | | | | |  | | | 0.394 |
| No | 0.35 | 0.08-1.63 | |  | | | 0.51 | | 0.11-2.38 | | |  | | 0.58 | | | | | 0.16-2.04 | | |  |

TNBC triple-negative breast cancer, ST special type, NST no special type, NAC neoadjuvant chemotherapy, OS overall survival, DDFS distant disease-free survival, DFS disease-free survival, pCR pathological complete response, CT chemotherapy, RT radiotherapy.
