# Supplementary material for: Prognostic impact of HER2-low expression in triple-negative breast cancer of high-grade special histological type and no special type
Source: PLoS One. 2025 Jun 13;20(6):e0325715. doi: 10.1371/journal.pone.0325715 (PMC12165359; doi:10.1371/journal.pone.0325715)
Supplement: S16 Table — (DOCX) [file pone.0325715.s016.docx]

**S16 Table. Univariate and multivariate analyses of clinicopathological variables in NAC-treated patients with HER2 1+/2+ high-grade TNBC ST and TNBC NST (n=61).**

| **Univariate** |  | **OS** |  |  | **DDFS** |  |  | **DFS** |  |
| --- | --- | --- | --- | --- | --- | --- | --- | --- | --- |
|  | **HR** | **95% CI** | ***p*-Value** | **HR** | **95% CI** | ***p*-Value** | **HR** | **95% CI** | ***p*-Value** |
| **Age** (years) |  |  |  |  |  |  |  |  |  |
| < 50 | 1 |  | 0.485 | 1 |  | 0.103 | 1 |  | 0.660 |
| ≥ 50 | 0.68 | 0.23-2.02 |  | 0.34 | 0.09-1.24 |  | 0.82 | 0.33-2.03 |  |
| **Year of diagnosis** |  |  |  |  |  |  |  |  |  |
| 2010-2017 | 1 |  | 0.543 | 1 |  | 0.440 | 1 |  | 0.630 |
| 2018-2023 | 0.70 | 0.22-2.23 |  | 0.65 | 0.22-1.94 |  | 0.79 | 0.29-2.10 |  |
| **TNBC subgroup** |  |  |  |  |  |  |  |  |  |
| NST | 1 |  | 0.493 | 1 |  | 0.760 | 1 |  | 0.631 |
| ST high-grade | 0.042 | 0.00-358.78 |  | 0.73 | 0.09-5.62 |  | 0.61 | 0.08-4.63 |  |
| **ypT category** |  |  |  |  |  |  |  |  |  |
| T0 | 1 |  | **<0.001** | 1 |  | **<0.001** | 1 |  | **<0.001** |
| T1/T2 | 4.41 | 0.51-37.98 |  | 4.01 | 0.45-35.98 |  | 4.39 | 0.94-20.55 |  |
| T3/T4 | 153.46 | 14.21-1657.09 |  | 142.94 | 13.00-1571.45 |  | 121.41 | 18.41-800.59 |  |
| **cT stage** |  |  |  |  |  |  |  |  |  |
| T1/T2 | 1 |  | **<0.001** | 1 |  | **<0.001** | 1 |  | **<0.001** |
| T3/T4 | 13.22 | 4.35-40.16 |  | 14.99 | 4.79-46.94 |  | 7.81 | 3.08-19.77 |  |
| **Nodal status** (post-NAC) |  |  |  |  |  |  |  |  |  |
| N- | 1 |  | **<0.001** | 1 |  | **<0.001** | 1 |  | **<0.001** |
| N+ | 12.13 | 3.37-43.67 |  | 12.23 | 3.35-44.59 |  | 5.08 | 2.03-12.67 |  |
| **Nodal status** (pre-NAC) |  |  |  |  |  |  |  |  |  |
| N- | 1 |  | **0.013** | 1 |  | **0.011** | 1 |  | **0.049** |
| N+ | 6.75 | 1.51-30.23 |  | 7.10 | 1.57-32.08 |  | 2.65 | 1.01-7.00 |  |
| **Grade** |  |  |  |  |  |  |  |  |  |
| G2 | 1 |  | 0.679 | 1 |  | 0.685 | 1 |  | 0.427 |
| G3 | 1.54 | 0.20-11.94 |  | 0.73 | 0.16-3.31 |  | 0.60 | 0.17-2.10 |  |
| **pCR** |  |  |  |  |  |  |  |  |  |
| Yes | 1 |  | **0.041** | 1 |  | 0.051 | 1 |  | **0.020** |
| No | 8.38 | 1.10-64.12 |  | 7.65 | 0.99-58.90 |  | 5.74 | 1.32-24.93 |  |
| **Adjuvant CT** |  |  |  |  |  |  |  |  |  |
| Yes | 1 |  | 0.835 | 1 |  | 0.954 | 1 |  | 0.263 |
| No | 0.88 | 0.27-2.85 |  | 0.97 | 0.30-3.14 |  | 0.58 | 0.22-1.52 |  |
| **Adjuvant RT** |  |  |  |  |  |  |  |  |  |
| Yes | 1 |  | 0.416 | 1 |  | 0.689 | 1 |  | 0.169 |
| No | 1.62 | 0.51-5.19 |  | 0.74 | 0.16-3.32 |  | 1.97 | 0.75-5.20 |  |
| **Multivariate** |  | **OS** |  |  | **DDFS** |  |  | **DFS** |  |
|  | **HR** | **95% CI** | ***p*-Value** | **HR** | **95% CI** | ***p*-Value** | **HR** | **95% CI** | ***p*-Value** |
| **Age** (years) |  |  |  |  |  |  |  |  |  |
| < 50 | - | - | - | 1 |  | 0.877 | - | - | - |
| ≥ 50 |  |  |  | 0.88 | 0.17-4.64 |  |  |  |  |
| **ypT category** |  |  |  |  |  |  |  |  |  |
| T0 | 1 |  | 0.093 | 1 |  | 0.587 | 1 |  | **0.031** |
| T1/T2 | 1.88 | 0.15-23.89 |  | 4.30 | 0.27-69.53 |  | 3.69 | 0.29-46.95 |  |
| T3/T4 | 27.54 | 0.96-792.38 |  | 417727.53 | - |  | 72.18 | 2.38-2191.31 |  |
| **cT stage** |  |  |  |  |  |  |  |  |  |
| T1/T2 | 1 |  | 0.750 | 1 |  | 0.937 | 1 |  | 0.953 |
| T3/T4 | 1.48 | 0.13-16.24 |  | 0.00 | - |  | 1.07 | 0.11-10.62 |  |
| **Nodal status** (post-NAC) |  |  |  |  |  |  |  |  |  |
| N- | 1 |  | 0.554 | 1 |  | 0.534 | 1 |  | 0.646 |
| N+ | 2.05 | 0.19-22.23 |  | 2.23 | 0.18-27.96 |  | 1.58 | 0.23-10.96 |  |
| **Nodal status** (pre-NAC) |  |  |  |  |  |  |  |  |  |
| N- | 1 |  | 0.805 | 1 |  | 0.592 | 1 |  | 0.692 |
| N+ | 1.37 | 0.11-16.33 |  | 2.02 | 0.15-26.72 |  | 1.40 | 0.26-7.52 |  |
| **pCR** |  |  |  |  |  |  |  |  |  |
| Yes | 1 |  | 0.366 | 1 |  | 0.931 | 1 |  | 0.862 |
| No | 3.12 | 0.27-36.50 |  | 1.13 | 0.07-18.35 |  | 1.25 | 0.10-15.49 |  |
| **Adjuvant RT** |  |  |  |  |  |  |  |  |  |
| Yes | - | - | - | - | - | - | 1 |  | 0.025 |
| No |  |  |  |  |  |  | 3.61 | 1.17-11.10 |  |

TNBC triple-negative breast cancer, ST special type, NST no special type, NAC neoadjuvant chemotherapy, OS overall survival, DDFS distant disease-free survival, DFS disease-free survival, pCR pathological complete response, CT chemotherapy, RT radiotherapy.
